# Yogurt Supplementation Can Ameliorate Fatty Liver Diseases and Metabolic Syndrome in High Fat‐Induced Conditions in Mice

**DOI:** 10.1002/fsn3.4650

**Published:** 2024-12-05

**Authors:** Sohel Hasan, Md Aminul Islam Amin, Masum Mia, Sumaiya Khatun, Yesir Arafat, Md Royhan Gofur, Md Mahmudul Islam, Md Eram Hosen, Khalid S. Almaary, Gezahign Fentahun Wondmie, Amirul Islam, Matiar Rahman, Mohammed Bourhia

**Affiliations:** ^1^ Molecular and Biomedical Research Lab (MBRL), Department of Biochemistry and Molecular Biology University of Rajshahi Rajshahi Bangladesh; ^2^ Department of Veterinary and Animal Sciences University of Rajshahi Rajshahi Bangladesh; ^3^ State Key Laboratory of Respiratory Disease, Guangzhou Institutes of Biomedicine and Health Chinese Academy of Sciences Guangzhou China; ^4^ Department of Microbiology, Shaheed Shamsuzzoha Institute of Biosciences Affiliated With University of Rajshahi Rajshahi Bangladesh; ^5^ Department of Botany and Microbiology, College of Science King Saud University Riyadh Saudi Arabia; ^6^ Department of Biology Bahir Dar University Bahir Dar Ethiopia; ^7^ Laboratory of Biotechnology and Natural Resources Valorization, Faculty of Sciences Ibn Zohr University Agadir Morocco

**Keywords:** hepatic steatosis, high‐fat diet, LD biogenesis, LD genes, lipid metabolism, therapeutic agents

## Abstract

Hepatic steatosis/non‐alcoholic fatty liver disease is a major public health delinquent caused by the excess deposition of lipid into lipid droplets (LDs) as well as metabolic dysregulation. Hepatic cells buildup with more fat molecules when a person takes high fat diet that is excessive than the body can handle. At present, millions of people in the world are affected by this problem. So, it is very important to know the effects of factors responsible for the disease. Here, the role of lipid droplet (LD) biogenesis and metabolism was analyzed and intended to investigate if defects in biogenesis/metabolic enzymes are responsible for the accumulation of lipids other than LDs in fatty liver disease in high‐fat‐induced conditions in mice model. To explore it, high‐fat diet (HFD), fast food (FF), and soft drinks (SD) were administered to wild‐type Swiss albino mice for 14 weeks following yogurt supplementation. After experimental period, glucose tolerance, enzyme function, lipid profile, plasma biochemistry, and other analytical tests were analyzed by auto‐analyzer including different oxidative stress markers. Lipids from hepatic tissues were extracted, and purified by Floatation Assay and subsequently analyzed by different biochemical and chromatographic techniques. Histological architecture of hepatocytes was performed using Zeiss microscope. Finally, increased amount of lipids biogenesis/accumulation was found in liver tissues that causes Fatty liver disease. Significantly, HFD, FF, and SD were identified as factors for the increased LD biogenesis and or lipid metabolic disorder. Nevertheless, yogurt supplementation can homeostasis those LD formation and metabolic syndrome as it increases the down regulation of lipid biogenesis as well as lipid metabolic rate. So, yogurt supplementation was considered as a novel agent for decreasing LD biogenesis as well as excessive accumulation of fat in hepatocytes which can be used as therapeutics for the treatment of NAFLD.

## Graphical abstract

Here, the role of lipid droplet (LD) biogenesis and metabolism was analyzed and intended to investigate if defects in biogenesis including metabolic enzymes are responsible for the accumulation of lipids other than LDs in the fatty liver especially in high‐fat diet (HFD), fast food (FF), and soft drinks (SD) mice model. To explore it, HFD, FF, and SD were administered to wild‐type Swiss albino mice for 14 weeks.

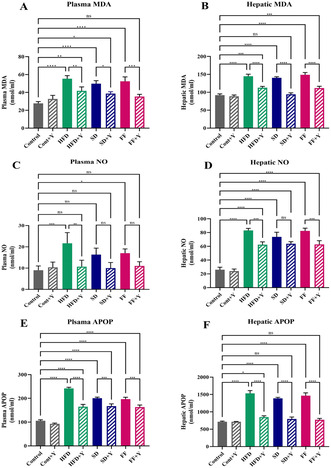

## 1 Introduction

Liver is indispensable for health as it is a central and major integrator of metabolism. Hepatic steatosis is caused by a variety of disorder that results abnormal accumulation of lipids in hepatic cells. Obesity‐related NAFLD manifestation by metabolic syndrome is a prevalent public health issue, has been consistently linked to a range of disorders comprising dyslipidemia, hypertension, resistance to insulin, and a greater vulnerability to cardiovascular mortality (Savini et al. 2013; Fazel et al. 2016). Obesity encompasses beyond simply being overweight, it involves the deposition of surplus calories from diets as visceral fat as well as the subsequent transport of concentrated free fatty acids (FAs) into multiple organs, ultimately triggering the onset of metabolic syndrome. The World Health Organization (WHO) classifies obesity as a “Medical condition whereby an excessive amount of fat develops to a degree that can have detrimental health effects”. A consequence thereof, persistent imbalance between calorie intake and expenditure eventually results in obesity, a chronic and heterogeneous illness. Furthermore, it is significantly impacted by behavioral, physiological, genetic, and external factors (Kaur 2014). At present, there is a worldwide overweight and obesity outbreak affecting people spanning all ages in countries both developed and developed. The global occurrence of obesity has had an exponential rise between the years 1980 and 2014. A report released by the WHO in 2015 displayed that, the worldwide fatality rate is higher for those who are considered overweight than for underweight people. So, it is an emerging public health concern that diminishes average lifespan and elevates mortality rates. Nevertheless, numerous research reports demonstrate that the global obesity crisis is exacerbated by things like high‐fat, high‐energy meals, and sedentary lifestyles (Park et al. 2010).

Excessive dietary intake, high accumulation of lipids in hepatic cells as well as increased biogenesis of lipids in lipid droplets and several diet‐related difficulties including metabolic syndrome and cardiovascular disease contribute to a condition called non‐alcoholic fatty liver disease (NAFLD) (Adolfsson, Meydani, and Russell 2004). The major lipid components are non‐polar lipids such as TAG and CEs that is stored in a dynamic organelle known as lipid droplets (LDs) in hepatocytes and in other cells including hepatic stellate cells (HSCs) and Kupffer cells. Fatty liver disease may be results by consuming high alcohols or non‐alcoholic with high fat diet induced conditions. Hepatic steatosis that is NAFLD is a widespread chronic hepatic condition that evolves in those who refrain from using alcohol. Numerous liver lesions, including severe fibrosis, cirrhosis, non‐alcoholic steatohepatitis (NASH), and basic steatosis, have been tied with NAFLD (Savini et al. 2013). Giving mice a diet enriched in fat and carbohydrates might lead them to display symptoms related to metabolic syndrome (Savini et al. 2013). Complex pathophysiological mechanisms have been identified in NAFLD, multifactorial disorders such as obesity, dyslipidemia, and insulin resistance (IR) are important clinical markers (Savini et al. 2013; Kaur 2014). However, pro‐inflammatory cytokines, oxidative stress, and aberrant hepatic lipid regulation all trigger the deposition of fat in liver tissues and cells like hepatic cells that is hepatocytes and primarily in HSCs. A number of research works pertaining to NAFLD relied on a combination of dietary carbohydrates and fats gathered from various sources, including organic ones (Park et al. 2010; Adolfsson, Meydani, and Russell 2004). Additionally, oxidative stress, which is correlated with obesity, is capable of causing numerous adverse effects such as nephropathy, microvascular difficulties, NAFLD, and endothelial dysfunction (Savini et al. 2013; Kaur 2014). Oxidative stress in the context of obesity may be attributed to several reasons and mechanisms of actions. The oxidation of FAs by peroxisomes and mitochondria, a high‐lipid diet, and increased demand for oxygen are some of the underlying roots. Obesity typically results in a decreased antioxidant enzymes level, such as glutathione peroxidase (GPx), catalase (CAT), and superoxide dismutase (SOD), which lessens an individual's antioxidant defenses in contrast to those who are of a normal weight. Antioxidant defense declines inversely proportionately to elevated central adiposity. Furthermore, high levels of reactive nitrogen species (RNS) or reactive oxygen species (ROS) are the signs that displayed and demonstrate of obesity. Previous research reported that obesity‐related hepatic inflammation may have an impact on the development of malignancies in mice that have been administered a diet aimed at encouraging obesity (Park et al. 2010). Prolonged NAFLD may result in severe hepatic damage, including liver fibrosis especially if it merges with other difficulties. In rare instances, there may be a correlation among other enzymes, notably COL4A1 and Col4A2 that can result in the onset of liver cancer (Liu et al. 2020).

Dairy products such as yogurt are produced when bacteria like *Lactobacillus bulgaricus* as well as other lactic acid bacteria (LAB) ferment the lactic acid in milk. It is widely acknowledged to be a high‐probiotic meal (Adolfsson, Meydani, and Russell 2004). Living bacteria identified as probiotics have favorable physiological impacts on their hosts. They assist in the production of antibacterial substances, stimulation of immunological reactions and a reduction of the pH level in the gastrointestinal tract (GIT) among additional health benefits (Abu‐Elsaad et al. 2015). As per prior research, consuming a probiotic pill may help replenish gut flora and provide another means of treatment for irritable bowel syndrome (Lee and Bak 2011; Hod et al. 2018). Probiotics also serve in the oversight of elevated blood cholesterol and diabetes (Kim et al. 2017). Through the degradation of cholesterol and deconjugation of bile salts, certain species of *Lactobacilli* and *Bifidobacterium* can lower cholesterol levels in experimental mice (Anderson and Gilliland 1999). Mice given an elevated fat diet exhibited a favorable change in the lipid profile after receiving a blend of probiotics containing *Lactobacillus* ( *L. reuteri* and *L. plantarum* ) and *Bifidobacterium* ( *B. longum* , *B. lactis* , and *B. breve* ) (Kim et al. 2017). Probiotics have also been found to aid people with NAFLD, in accordance with a recent meta‐analysis (Lavekar et al. 2017). Additionally, research indicates that probiotics, which are found in yogurt, have an advantageous impact on cholesterol burn/metabolism and liver enzymes activity levels that improve NAFLD conditions (Bakhshimoghaddam et al. 2018; Nabavi et al. 2014).

Nevertheless, the precise means by which probiotics improved NAFLD and reduced oxidative stress in the liver remain unclear. This investigation examined the impacts of incorporating raw yogurt supplement with vit‐B complex on the development of fatty liver disease, fat accumulation, blood lipid levels and lipid droplet formation in mice administered a diet heavy in fast food and soft drinks, outlining the positive effects of this functional food item was the aim. Hence, analyzing the lipid molecules with its metabolic syndrome and identification of yogurt as important therapeutic agents for fatty liver disease was the main focus.

## 2 Materials and Methods

### 2.1 Reagents and Chemicals

We bought tiobarbituric acid (TBA) from Sigma‐Aldrich in Germany. J.I. Baker provided the reduced glutathione (GSH) that was needed. (USA). DCI Diagnostics (Budapest, Hungary) provided the aspartate aminotransferase (AST), alanine aminotransferase (ALT), alkaline phosphatase (ALP), triglyceride, cholesterol, HDL, and LDL assay kits. The remaining substances, solvents, and reagents utilized in this investigation were all of laboratory analytical quality.

### 2.2 Preparation of Yogurt

This yogurt is made up of cow's milk, skimmed milk powder and bacterial cultures. Mainly two bacterial cultures were used to prepare the yogurt, such as *Streptococcus lactis* and *Lactobacillus bulgaricus* . Yogurt also contains 3.2 g fat, 5.03 g carbohydrate, 5.67 g protein and 169 mg calcium in 100 g which can provide 71.6 kcal energy. In general, collected milk was pasteurized first at 90°C for 16 s and then transferred to yogurt milk processing tank where bacterial cultures were added at 41°C–43°C temperature. This processed milk is then transferred to small containers where the yogurt was appeared at incubation temperature of 43°C. The prepared yogurt containers were kept under 4°C temperature in storage condition.

### 2.3 Experimental Animals and Treatment in High Fat Induced Condition

All experimental protocols for animal care, handling, and experimentation (249(35)/320/IAMEBBC/I.B.Sc.) were approved by the Ethics Committee of Institute of Biological Sciences, University of Rajshahi, Bangladesh. In addition, we would like to certify that every experiment was carried out in compliance with all applicable laws and rules 48 male Swiss albino mice, aged between 5 and 7 weeks and weighing between 22 and 25 g, were acquired from the Animal Reproduction Unit of the I.C.D.D.R.B. in Dhaka, Bangladesh. Additionally, they were kept in separate cases in a 22°C ± 2°C, 55% humidity room with a 12‐h light/dark cycle, and they had free access to purified water and laboratory feed.

In this investigation, standard laboratory foods included a high‐fat Diet (HF), fast food (FF), and soft drink (SD). The typical chow diet for control mice consisted of wheat, rice polishing, wheat bran, and fish meal, with a caloric value of roughly 25% proteins, 60% carbohydrates, and 15% fat (Rahman et al. 2017). For experimental mice, High Fat Diet (Custom diet for lab animal, 60% fat, SYNERGY BIO, China) was administered. High‐Fat (HF) diet included normal foods such as sugar, condensed milk, and beef tallow, and its calorie composition was roughly 14% proteins, 37% carbs, and 60% fat. FF contains normal food with 30% cheese, 20% mayonnaise and 10% tomato slouch. SDs contain 2 mL Coca‐Cola daily intake by the gavage.

The eight groups of experimental mice for the period of 14 weeks, each group with six mice, were as follows:

- (Group 1) Control, received normal food (powder) and water.
- (Group 2) High fat (HF), received the HF diet.
- (Group 3) Control + yogurt, received normal food (powder) and water along with yogurt (10% w/w).
- (Group 4) High fat (HF) + yogurt, received the HF diet and yogurt (10%, w/w).
- (Group 5) Soft drink (SD) + yogurt, received normal food (powder) and water along with one time Coca‐Cola soft drinks with yogurt (10% w/w).
- (Group 6) Soft drink (SD), received normal food (powder) and water with one time Coca‐Cola soft drinks.
- (Group 7) Fast food (FF), received normal food (powder) and water along with Fast‐food ingredient.
- (Group 8) Fast Food (FF) + yogurt, received normal food (powder) and water along with fast‐food ingredient and yogurt (10%, w/w).

To measure the glycemic activity before and after consuming the HF diet, an OGTT was administered to mice in each of the eight groups both before and after the experimental feeding period.

### 2.4 Experimental Animal Sacrifice and Sample Collection

During the experiment feeding period (98 days), body weight, food consumption, and water intake were recorded daily. After the experiment, all the mice were weighed and sacrificed by injecting pentobarbital anesthesia (80 mg/kg body weight) in the peritoneal region. Samples of blood were drawn from the abdominal aorta and placed in tubes with citrate buffer at 4°C. Blood samples were centrifuged at 8000 rpm for 15 min at 4°C within 30 min of blood collection in order to separate the plasma. The separated plasma was then placed in 1.5 mL Eppendorf tubes and kept cold until additional examination. Upon animal sacrifice, all other internal organs such as the liver, spleen, intestine, heart, and adipose tissues were promptly removed and preserved in neutral buffered formalin (pH‐7.4) for histological examinations. Additionally, the organs were weighed and kept at −20°C for additional biochemical research.

### 2.5 Oral Glucose Tolerance Test (OGTT)

All groups took the oral glucose tolerance test at the start of the investigation, and then they fasted for 12 h. A commercial glucometer (Accu Chek Instant S) was used to measure the blood glucose level in all of the mice during their fast. The glucose solution was given at a dose of 2 g/kg, and the blood glucose level was checked at 30, 60, 90, and 120 min. Following a 12‐h fast, the mice were fed for 98 days, and at the conclusion of this time, an oral glucose tolerance test was conducted in accordance with earlier research findings (Ulla et al. 2017).

### 2.6 Plasma Biochemistry

Collecting blood samples from every mouse slain in less than 30 min, followed by a 15‐min centrifugation at 8000 rpm and 4°C Before analysis, the plasma was separated, put into 1.5 mL Eppendorf tubes, and kept at −20°C. Using Diatec diagnostic kits (Hungary), the concentration of total cholesterol, HDL, LDL, and triglycerides as well as the activities of aspartate transaminase (AST), alanine transaminase (ALT), and alkaline phosphatase (ALP) were measured in accordance with the standards and protocols supplied by the manufacturer.

### 2.7 Preparation of Hepatic Tissue for Lipid Extraction and Analysis

The Bligh and Dyer (Bligh and Dyer 1959) method was used to assess the lipid content of the liver of mice. About 10 mL of distill water were used to ground 2 g of mice liver. After the flesh was ground, it was placed in a separating funnel and 30 mL of a 1:12 V/V chloroform–methanol combination were added. It was then stored in the dark at room temperature for the entire night. After this time, 20 mL of chloroform and 20 mL of water were added and combined. After being meticulously gathered in a 50 mL beaker that had been previously weighed, the chloroform layer was placed on a steam bath to evaporate. Following the chloroform's evaporation, the beaker's weight was once more measured. The amount of lipid is shown by the weight difference.

### 2.8 Thin Layer Chromatography (TLC) Analysis of Hepatic Lipid

Lipids were extracted by the Bligh and Dyer method (Bligh and Dyer 1959). The solvent was eliminated by vacuum‐assisted evaporation after the organic layer had been cleaned three times with 1 M KCl. Lipids were extracted from the LDs of different group of mice liver (2 g) with the same wet weight of cells. For non‐polar lipids, *n*‐hexane:diethyl ether:acetic acid (80:20:1) was developing solvent. For polar lipids, the solvent system was chloroform:methanol:acetone:acetic acid:water (65:35:10:5:4) as developing solvent. Prior to use, the plates were dried and activated at 110°C for 1 h (Stahl 1969). Lipid molecules were visualized by iodine vapor and were identified according to TLC standard 18‐5A marker. The *R*_*f*_ values were calculated and llipid bands were digitally scanned and analyzed the densitometry by using Image J. Characterization was performed in order to find out the significance of values. The iodine value, acid value, peroxide value and saponification value were determined by using (Hanus Method et al., 1996; IUPAC 1977; IUPAC 1977; AOCS 1998 and conventional procedure IUPAC 1977), respectively. The saponification value was used to determine the saponification equivalent. The acid value was used to compute the FFA %. Calculating the amount of unsaponifiable stuff required Hilditch (Hilditch TP 1949) formula was used.

### 2.9 Preparation of Tissue Samples

Liver tissue was homogenized in 10 mL of phosphate buffer containing (pH‐7.4) and spun at 8000 rpm for 15 min at 4°C in order to estimate the oxidative stress marker. After the supernatant was collected, it was used for enzymatic and protein analysis.

### 2.10 Analysis of Oxidative Stress Markers

#### 2.10.1 Estimation of Lipid Peroxidation

By using a previously reported approach, thiobarbituric acid reactive substances (TBARS) were measured calorimetrically to quantify lipid peroxidation in the liver (Niehaus Jr. and Samuelsson 1968). 0.1 mL of tissue homogenate (Tris–Hcl buffer, pH 7.5) was used to determine the amount of lipid peroxidation in the sample. This was followed by treatment with 2 mL of the 1:1:1 ratio TBATCA‐HCl reagent (thiobarbituric acid 0.37%, 0.25 N HCl, and 15% TCA). After that, the solution was transferred to a sealed Eppendorf tube, heated for 15 min in a hot water bath, and allowed to cool to room temperature. At 535 nm, the absorbance of the clear supernatant was measured in comparison to the reference blank. A straight‐line equation for the MDA standard curve was used to measure the concentration of MDA. The MDA concentration was given in either nmol/g or nmol/mL of the tissues.

#### 2.10.2 Assay of Nitric Oxide (NO)

Using Griess reagents, nitrate (NO) was measured using the procedure outlined by Tracey (Tracey, Tse, and Carter 1995). Instead of utilizing 1‐napthylamine (5%), naphthyl ethylene diamine di‐hydrochloride (0.1% w/v) was used in this investigation to modify the Griess‐Ilosvay reagent. The reaction mixture (3 mL) was incubated with tissue homogenates (2 mL) and phosphate buffer saline (0.5 mL) at 25°C for 150 min. A chromophore with a pink hue developed. These solutions' absorbance was measured at 540 nm in comparison to the matching blank solutions. The NO level was measured in nmol/mL or nmol/g of tissue using a standard curve.

#### 2.10.3 Advanced Oxidation Protein Products (APOP) Assay

With some modifications, the methods of Witko and Tiwari (Witko‐Sarsat et al. 1996; Tiwari et al. 2014) were modified to determine the APOP level. PBS was used to dilute 2 mL of plasma 1: 5. After 2 min, 0.2 mL of acetic acid and 0.1 mL of potassium iodide (1.16 M) were added to each tube. At 340 nm, the absorbance of the reaction mixture was measured right away against a blank that included 0.2 mL of acetic acid, 0.1 mL of KI, and 2 mL of PBS. The absorbance of chloramine‐T at 340 nm was discovered to be linear between 0 and 100 nmol/mL. The concentrations of AOPP were represented in terms of nmol mL^−1^ chloramine‐T equivalents.

### 2.11 Assay of the Activities of the Antioxidant Enzyme

#### 2.11.1 Catalase Activity Assay (CAT)

The approach by Chance and Maehly (1955) previously outlined was used to determine the CAT activities. 2.5 mL of 50 mmol phosphate buffer (pH 5.0), 0.4 mL of 5.9 mmol H_2_O_2_, and 0.1 mL of tissue homogenates made up the CAT activity reaction solution. After a minute, the reaction solution's changes in absorbance at 240 nm were measured. A 0.01 units/min change in absorbance was used to define one unit of CAT activity.

#### 2.11.2 Superoxide Dismutase (SOD) Activity Assay

Using a previously reported method, SOD was measured in plasma and tissue homogenates (Misra and Fridovich 1972). An aliquot of tissue homogenates and PBS were added to the 3 mL reaction mixture to bring the total volume to 2.94 mL. The injection of 0.06 mL of 15 mM epinephrine initiated the process. For 1 min, at 15‐s intervals, the absorbance change at 480 nm was measured. All ingredients except for tissue homogenates were used in a control that was run concurrently. The auto‐oxidation of the epinephrine in the assay system is defined as 50% inhibition at one unit of enzyme activity.

#### 2.11.3 Reduced GSH Activity Assay

We estimated reduced GSH using the Jollow (Jollow et al. 1974) approach. One milliliter (1.0 mL) of 4% sulfosalicylic acid was added to the homogenate of tissue. After being stored at 4°C for 1 h, the samples were centrifuged at 1200 × *g* for 20 min at 4°C. 0.1 mL tissue homogenate, 2.7 mL phosphate buffer (0.1 M, pH 7.4), and 0.2 mL DTNB (5,5‐dithiobis‐2‐nitrobenzoic acid) (100 mM) made up the entire amount of the 3.0 mL assay mixture. The combination took on a yellow hue, which was measured right away at 412 nm and reported as ng/mg protein.

#### 2.11.4 Estimation of Myeloperoxidase (MPO) Activity Assay

A dianisidine‐H_2_O_2_ technique by Bradley et al. (1982) that was adjusted for 96‐well plates was used to measure MPO activity. In summary, 50 mM potassium phosphate buffer (pH 6.0) containing 0.53 mM o‐dianisidine di‐hydrochloride (Sigma) and 0.15 mM H_2_O_2_ were combined with 10 μg protein plasma sample added in triplicate. At 460 nm, the absorbance change was observed. The results were given in MPO/mg protein units.

### 2.12 Histopathological Analysis

Liver tissues were embedded in paraffin, fixed in neutral buffered formalin, sectioned at 5 μm, and then stained with hematoxylin/eosin for microscopic examination in order to observe the architecture of the liver tissue and inflammatory cell infiltration. After that, sections were examined and captured on camera at 10× and 40× magnification using a light microscope (Zeiss Axioscope).

### 2.13 Staining With Oil Red O for Histochemistry Analysis of LDs

Selected liver samples were fixed in 10% neutral buffered formalin and embedded in paraffin, then cut at 6 μm thickness using microtome, and sliced sections were stained with hematoxylin and eosin. Frozen sections were stained with Oil red O (working solution of Oil red O in isopropanol) for histochemistry analysis of lipid droplet in hepatocytes. After staining, the sections were rehydrated in descending grades of alcohol, cleared in xylene and mounted with “DPX”. The stained sections of liver of different treated groups were studied thoroughly under compound microscope using 10 and 40 objectives (Model B‐293PLi, OPTIKA, Italy).

### 2.14 Statistical Analysis

Data are presented as mean ± SD, *n* = 6. Statistical analysis was performed by One Way ANOVA with Šídák post hoc test using GraphPad Prism (10.2.3(403)). In every instance, a significance level of *p* < 0.05 was taken into account.

## 3 Results

### 3.1 Effect of Food, Water Intake and Yogurt on Body Weight in High Fat Induced Conditions

High fat diet had a predominant role on obesity that causes higher body weight gain as well as dysregulation of metabolic syndrome. Diet heavy in fat and energy was thought to be a major contributing factor to the development of these problems finally tends to be in hepatic steatosis. During the experimental period, the body weight of each mouse was recorded daily where HFD, FF, and SD diet‐treated mice showed a significant increase in body weight as compared to the control group (Table 1). Subsequently, supplementing with yogurt reduced the body weight gain in HFD + Y, FF + Y and SD + Y diet feed mice, respectively (Figure S2). Moreover, water and food consumptions were also reduced in HFD, FF, SD, HFD + yogurt, FF + yogurt, and SD + yogurt mice compared to control and Control + yogurt mice, respectively. However, in contrast to the control mice, the HFD diet‐fed mice consumed more energy (Table 1). So, HFD, SD, and FF diet causes the increased in body weight, whereas yogurt supplementation significantly reduces the weight gain. Here, these effects might be due to the role of yogurt that might play an important role by upregulating the metabolic rate of lipids and energy homeostasis by stimulating the immunological reactions.

**TABLE 1:** Effect on mice body weight, food intake, and water intake during treatment period.

Group	Initial body weight (g)	Final body weight (g)	Food intake (g/day)	Water intake (mL/day)	Calorie intake (kj/day)
Control	27.67 ± 2.51	41.7 ± 1.10	15.23 ± 0.65	12.13 ± 0.40	406 ± 4.00
Control + Y	30 ± 2.00	39.47 ± 1.75	14.63 ± 0.50	11.4 ± 0.55	401.33 ± 3.50
HFD	29.33 ± 2.51	49.57 ± 0.70	13.86 ± 0.45	11.4 ± 0.36	676 ± 4.00
HFD + Yogurt	29 ± 2.00	43.06 ± 0.75	14.70 ± 0.66	11.23 ± 0.50	627 ± 6.55
SD	28.7 ± 2.51	47.4 ± 0.91	13.73 ± 0.56	11.8 ± 0.36	649.67 ± 3.51
SD + Yogurt	28.33 ± 3.51	42.43 ± 0.70	14.73 ± 0.35	11.2 ± 0.3	613.33 ± 5.50
FF	29 ± 2.00	49.2 ± 1.30	13.83 ± 0.35	11.4 ± 0.4	669 ± 3.00
FF + Yogurt	30 ± 2.00	43.53 ± 0.80	14.36 ± 0.50	11.56 ± 0.30	625.67 ± 3.05

*Note:* Comparison of the effects of yogurt supplementation to a control, HFD, SD, and FF diet on body weight, food consumption, water intake, and total calorie intake. Control mice were provided with chow diet, FF mice were provided with FF diet, SD mice were provided with Coca‐Cola SD and HFD mice were provided with high fat diet, respectively. Yogurt was also supplied to control + yogurt, FF + yogurt, SD + yogurt, and HF + yogurt groups. Data are presented as mean ± SD, *n* = 6. Statistical analysis was performed by GraphPad Prism (10.2.3(403)).

### 3.2 Effect of Yogurt Supplementation on Oral Glucose Tolerance Test (OGTT)

**FIGURE 1:**
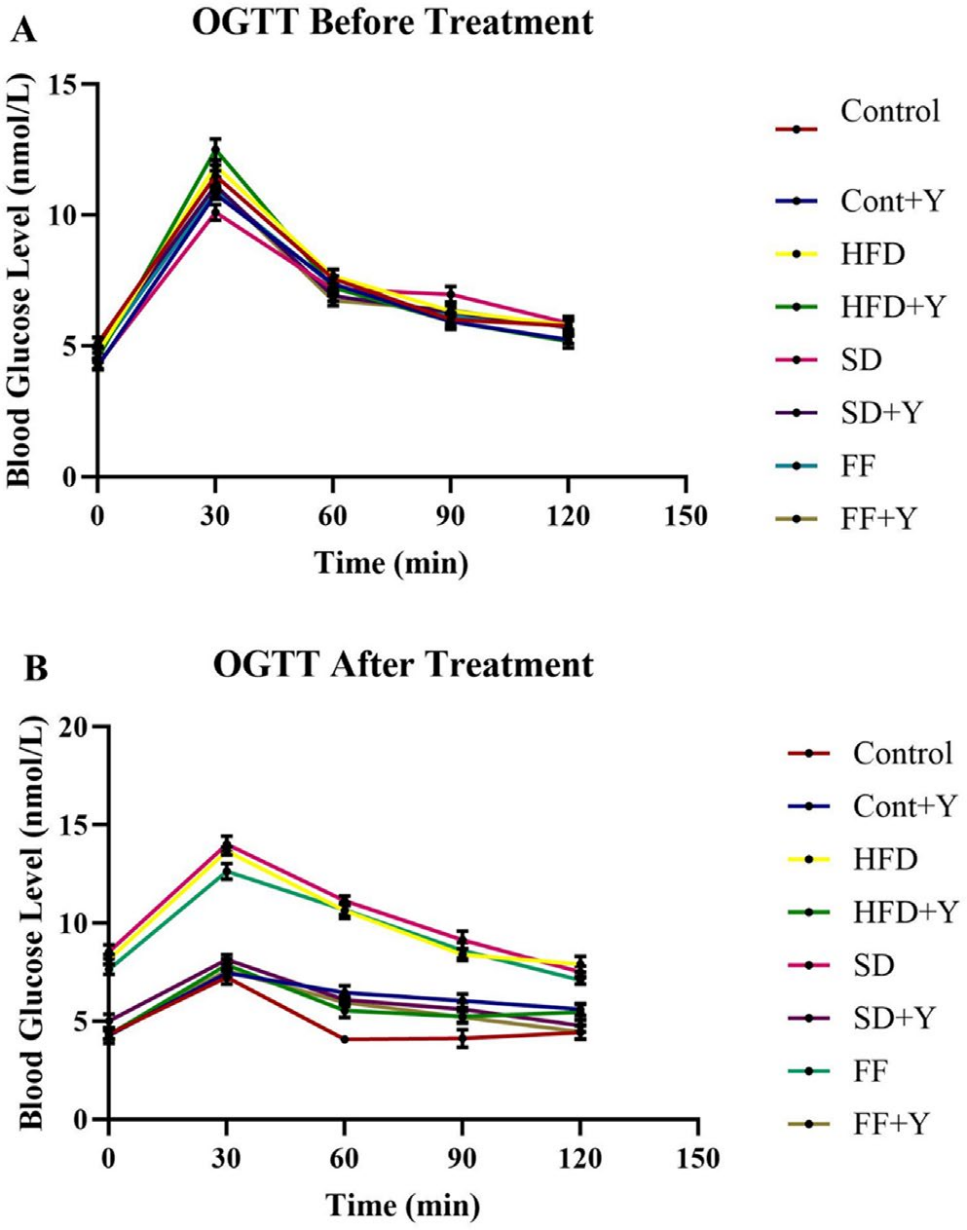
Effect of yogurt supplementation on oral glucose tolerance test (OGTT) before and after HFD, FF, and SD diet‐fed mice. Control mice were provided with control diet and HFD, FF and SD group were provided with HF diet, FF diet and SD diet as Coca‐Cola. Yogurt was provided to the HFD + yogurt, SD + yogurt, FF + yogurt, and control + yogurt groups, respectively. (A) OGTT was conducted at the start of the study, and (B) OGTT was completed at its conclusion. Data are presented as mean ± SD, *n* = 6. Statistical analysis was performed by using GraphPad Prism (10.2.3(403)).

The uptake of glucose and its metabolism is an important factor for diabetes as well as obesity. In high‐fat‐induced conditions, along with NAFLD, dysregulation of glucose metabolism might be occurred. The outcomes of oral glucose tolerance test of the both experimental and control obese mice are demonstrated in Table 2. In mice that were given a normal glucose load, the blood glucose level peaked 60 min after the glucose was added, and it then dropped to almost normal levels 120 min later (Figure 1). In contrast, in obese mice that were given an HFD, FF, or SD diet, the peak rise in blood glucose levels was seen 60 min later and continued to rise for the next 60 min. It is interesting to noted that yogurt supplementation to obese mice resulted in a considerable drop in blood glucose levels at 60 min and beyond, when compared to HFD, FF and SD diet and control mice (Figure 1). This finding strongly suggested that yogurt supplementation normalized the glucose metabolism that helps to prevent obesity and diabetes.

**TABLE 2:** Effect of yogurt on oral glucose tolerance test (OGTT).

	0 min	30 min	60 min	90 min	120 min
OGTT before treatment					
Control	5.10 ± 0.200	11.50 ± 0.400	7.57 ± 0.351	6.00 ± 0.264	5.76 ± 0.251
Control + Y	4.23 ± 0.152	10.80 ± 0.200	7.36 ± 0.300	5.93 ± 0.300	5.23 ± 0.152
HFD	4.73 ± 0.251	11.90 ± 0.200	7.66 ± 0.251	6.33 ± 0.251	5.80 ± 0.200
HFD + Y	4.53 ± 0.404	12.50 ± 0.400	7.23 ± 0.305	5.93 ± 0.251	5.16 ± 0.251
SD	4.30 ± 0.200	10.10 ± 0.300	7.16 ± 0.251	6.96 ± 0.305	5.86 ± 0.251
SD + Y	5.03 ± 0.305	11.13 ± 0.251	6.90 ± 0.200	6.30 ± 0.200	5.70 ± 0.264
FF	4.73 ± 0.251	10.96 ± 0.305	7.30 ± 0.200	6.13 ± 0.251	5.76 ± 0.351
FF + Y	4.76 ± 0.251	11.10 ± 0.360	6.73 ± 0.208	6.36 ± 0.305	5.70 ± 0.264
OGTT after treatment					
Control	4.33 ± 0.251	7.23 ± 0.351	4.06 ± 0.152	4.13 ± 0.450	4.43 ± 0.351
Control + Y	4.26 ± 0.404	7.46 ± 0.251	6.46 ± 0.351	6.03 ± 0.351	5.60 ± 0.300
HFD	8.16 ± 0.251	13.66 ± 0.208	10.63 ± 0.404	8.40 ± 0.300	7.90 ± 0.400
HFD + Y	4.30 ± 0.200	7.86 ± 0.305	5.53 ± 0.351	5.23 ± 0.305	5.46 ± 0.404
SD	8.53 ± 0.351	14.03 ± 0.416	11.13 ± 0.251	9.13 ± 0.450	7.50 ± 0.300
SD + Y	5.00 ± 0.360	8.13 ± 0.251	6.10 ± 0.200	5.60 ± 0.360	4.76 ± 0.321
FF	7.63 ± 0.251	12.63 ± 0.404	10.66 ± 0.251	8.60 ± 0.400	7.10 ± 0.200
FF + Y	4.33 ± 0.251	7.56 ± 0.450	5.96 ± 0.208	5.20 ± 0.300	4.46 ± 0.351

*Note:* OGTT of all groups were measured at initial stage before the experiment conducted and after 98 days of experimental period. All groups were treated with the glucose solution at a dose of 2 g/kg, and the blood glucose level was checked at 30, 60, 90, and 120 min. A commercial glucometer (Accu Chek Instant S) was used to measure the blood glucose level in all of the mice during their fasting state after the treatment.

### 3.3 Effects of HFD and Yogurt Supplementation on Liver Wet Weight, Fat Pad Deposition and Adipose Tissue Weights

**FIGURE 2:**
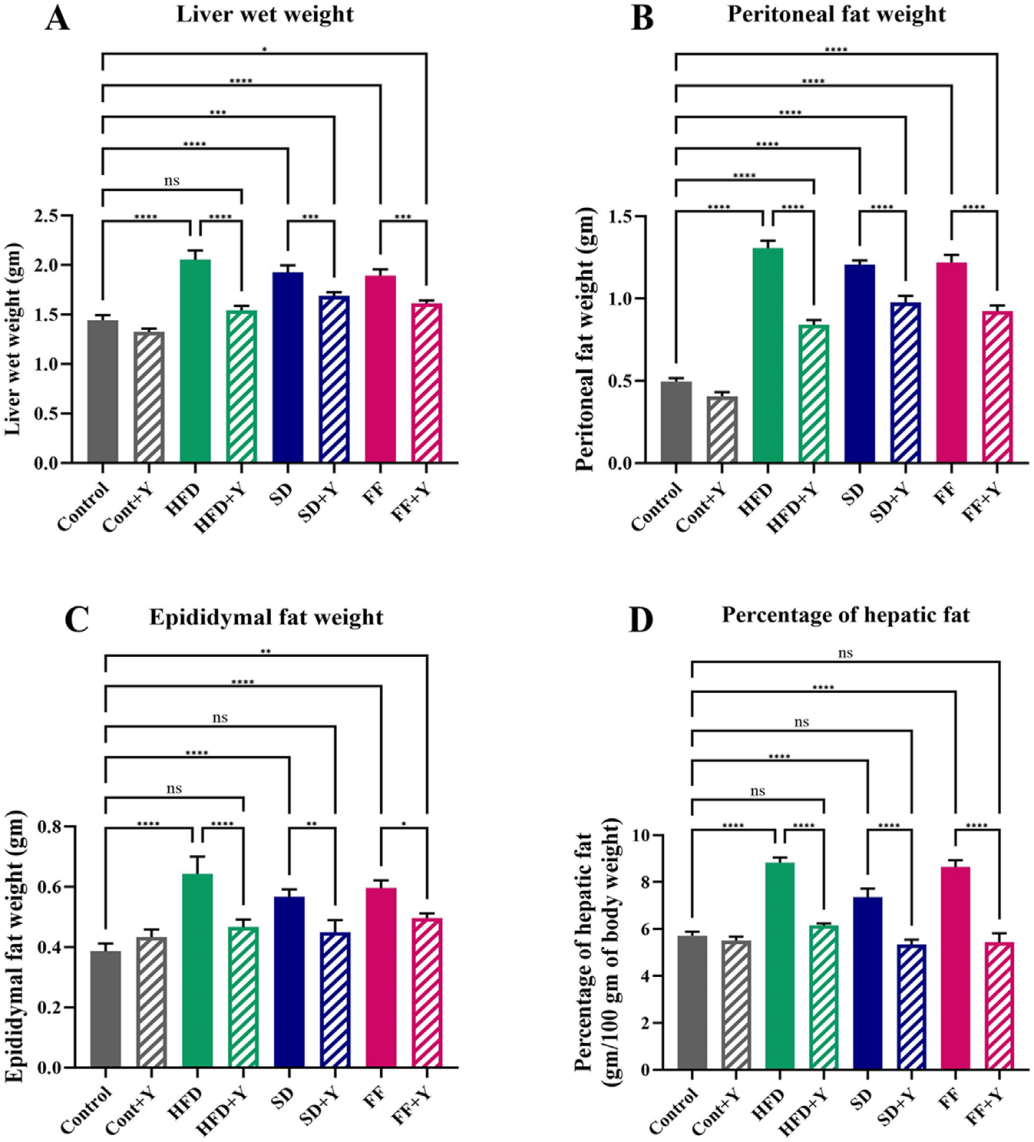
Effect of yogurt supplementation on; (A) liver wet weight, (B) peritoneal fat weight, (C) epididymal fat weight, and (D) percentage of hepatic fat of HFD, SD, and FF‐treated mice. Control mice were provided with control diet and HFD, FF, and SD were provided with HF diet, FF diet, and Coca‐Cola. Yogurt was also provided to control + yogurt and HFD + yogurt, FF + yogurt, and SD + yogurt groups. Data are presented as mean ± SD, *n* = 6. Statistical analysis was performed by One Way ANOVA with Šídák post hoc test using GraphPad Prism (10.2.3(403)). Statistical significance is considered as *p* < 0.05. Asterisk (****) marked data are significantly different at *p* < 0.0001 and (ns) denotes *p* > 0.05.

High‐fat‐induced condition enables and generated the fatty liver with higher amount of weight gain as well as fat pad deposition in tissues and organs like adipose tissue. Table 3 demonstrates the effects of different treatments of HFD, FF, SD diet, and yogurt, respectively on the mice liver or especially the weight gain and increased lipid/fat accumulation in obese condition. It displayed very important findings of liver wet weight in high‐fat‐induced conditions which was much higher than control group (Figure 2). Subsequently, Table 3 illustrated the significant reduction in wet weight of liver and fat pad deposition caused by yogurt supplementation in mice fed with the HFD, SD, and FF diets. The liver wet weight of the mice on a HFD, FF, and SD was considerably (*p* < 0.05) higher than that of the control group. Consequently, mice treated with HFD, FF, and SD, the wet weight of the liver was considerably (*p* < 0.05) reduced by supplementing with yogurt (5% w/w of diet). Significant variations were reported in the amounts of fat pad deposition among the control, and control + yogurt supplemented, HFD, HFD + yogurt, FF, FF + yogurt, SD, and SD + yogurt groups (Figure 2). In comparison to control mice, the wet weights of mesenteric, retroperitoneal, and epididymal adipose fat pads were significantly higher in mice fed with HFD, FF, and SD diets. This might be due to the abnormal lipid accumulation in hepatocytes in which the increased biogenesis of lipid molecules in lipid droplets occurred and results in dysregulation of lipid metabolism.

**TABLE 3:** Effect of yogurt supplementation on liver and adipose tissue weight.

Group	Liver wet weight (g)	Peritoneal fat weight (g)	Epididymal fat weight (g)	Mesenteric fat weight (g)	Percentage of hepatic fat
Control	1.443 ± 0.050	0.497 ± 0.021	0.387 ± 0.025	0.273 ± 0.021	5.713 ± 0.175
Control + Y	1.324 ± 0.033	0.407 ± 0.025	0.433 ± 0.025	0.307 ± 0.021	5.500 ± 0.171
HFD	2.053 ± 0.093	1.307 ± 0.045	0.643 ± 0.057	0.470 ± 0.040	8.827 ± 0.232
HFD + Yogurt	1.543 ± 0.043	0.840 ± 0.030	0.467 ± 0.025	0.313 ± 0.025	6.153 ± 0.085
SD	1.924 ± 0.073	1.207 ± 0.025	0.567 ± 0.025	0.407 ± 0.025	7.353 ± 0.365
SD + Yogurt	1.687 ± 0.038	0.977 ± 0.040	0.450 ± 0.040	0.327 ± 0.025	5.337 ± 0.210
FF	1.894 ± 0.061	1.220 ± 0.046	0.597 ± 0.025	0.410 ± 0.030	8.640 ± 0.298
FF + Yogurt	1.613 ± 0.030	0.923 ± 0.035	0.497 ± 0.015	0.290 ± 0.020	5.440 ± 0.383

*Note:* Effect of yogurt supplementation on liver weight, peritoneal fat weight, epididymal fat weight, mesenteric fat weight, and percentage of hepatic fat. Control mice were provided with control diet fast food mice were provided with fast food diet, soft drink mice were provided with Coca‐Cola soft drink and HF mice were provided with HF diet. Yogurt was also supplied to control + yogurt, FF + yogurt, SD + yogurt, and HF + yogurt groups. Data are presented as mean ± SD. *n* = 6. Statistical analysis was performed by GraphPad Prism (10.2.3(403)).

### 3.4 Effect of Yogurt Supplementation on Serum ALT, AST and ALP Activities

**FIGURE 3:**
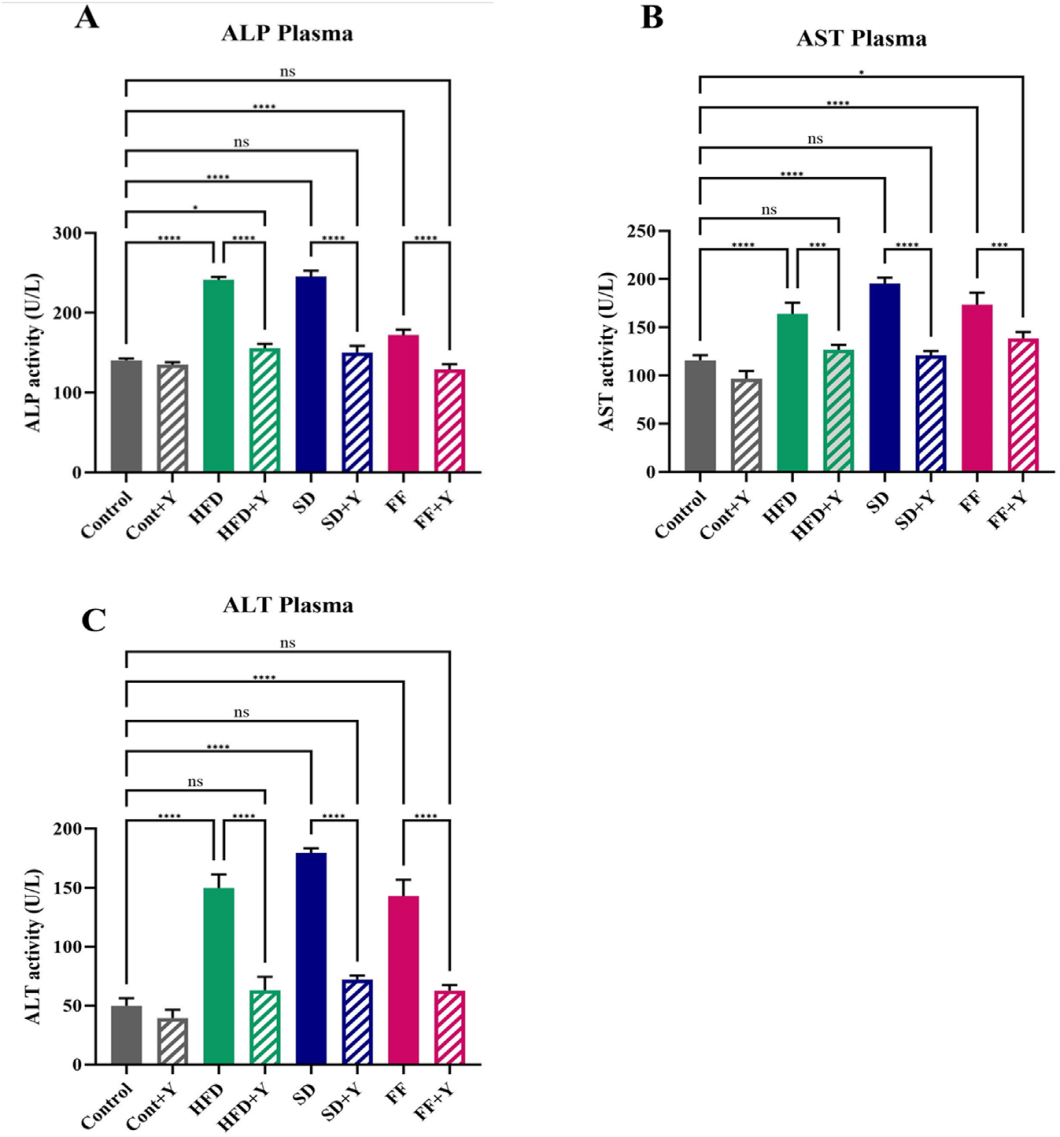
Effect of yogurt supplementation on liver Enzyme marker; (A) ALP, (B) AST and (C) ALT of HFD, SD, and FF‐treated mice. Control mice were provided with control diet and HFD, FF and SD were provided with HF diet, FF diet, and Coca‐Cola. Yogurt was also provided to control + yogurt, HFD + yogurt, FF + yogurt, and SD + yogurt groups. Data are presented as mean ± SD, *n* = 6. Statistical analysis was performed by One Way ANOVA with Šídák post hoc test using GraphPad Prism (10.2.3(403)). Statistical significance is considered as *p* < 0.05. Asterisk (****) marked data are significantly different at *p* < 0.0001 and (ns) denotes *p* > 0.05.

ALT (alanine transaminase), AST (aspartate aminotransferase) and ALP (alkaline phosphatase) are thought to be indicators of hepatic dysfunction and found high in liver injury, liver damage or liver disease. These enzymes are typically transferred to the plasma as a result of hepatocyte injury. On the other hand, the increased plasma activity of the AST, ALT, and ALP enzymes show that oxidative stress in tissues also causes hepatocyte injury in mice fed a HFD, FF, and SD diet. Here, mice fed with HFD, FF, or SD diets had higher plasma ALP, ALT, and AST activity along with increased liver wet weights compared to control mice (Figure 3). This is because of abnormal functions of liver enzymes that caused by the fatty liver disease. On the other hand, yogurt supplementation to HFD, FF and SD diet‐treated mice restored the normal liver function enzyme activities, as seen by a decrease in the plasma activities of the enzymes ALT, AST, and ALP (Figure 3). In hepatic steatosis conditions, ALT levels become high due to abnormal function of liver due to inflammation or hepatic injury. ALT is the most widely used single liver chemistry employed in the diagnosis and utmost important tools commonly used for detecting NAFLD, which was decreased in the case of yogurt supplementation. This might be due to the metabolic activity of probiotic effects of yogurt that normalize the ALT activity, which is important for normal function of liver. Yogurt is helpful in reducing intestinal and liver inflammation and function in decreasing hepatic injury to maintain normal liver condition. This helpful activity of yogurt to these enzymes thereby reduces the magnitude of fatty liver disease NAFLD. This is a very significant findings of the identifications of factors causing the liver disease as well as its therapeutic agents. Hence, yogurt significantly helps liver to boost up enzymes function for metabolism which is very much important to combat against fatty liver disease.

### 3.5 Effects of Yogurt Supplementation on Plasma and Liver Antioxidant Capacity and Lipid Peroxidation Markers

**FIGURE 4:**
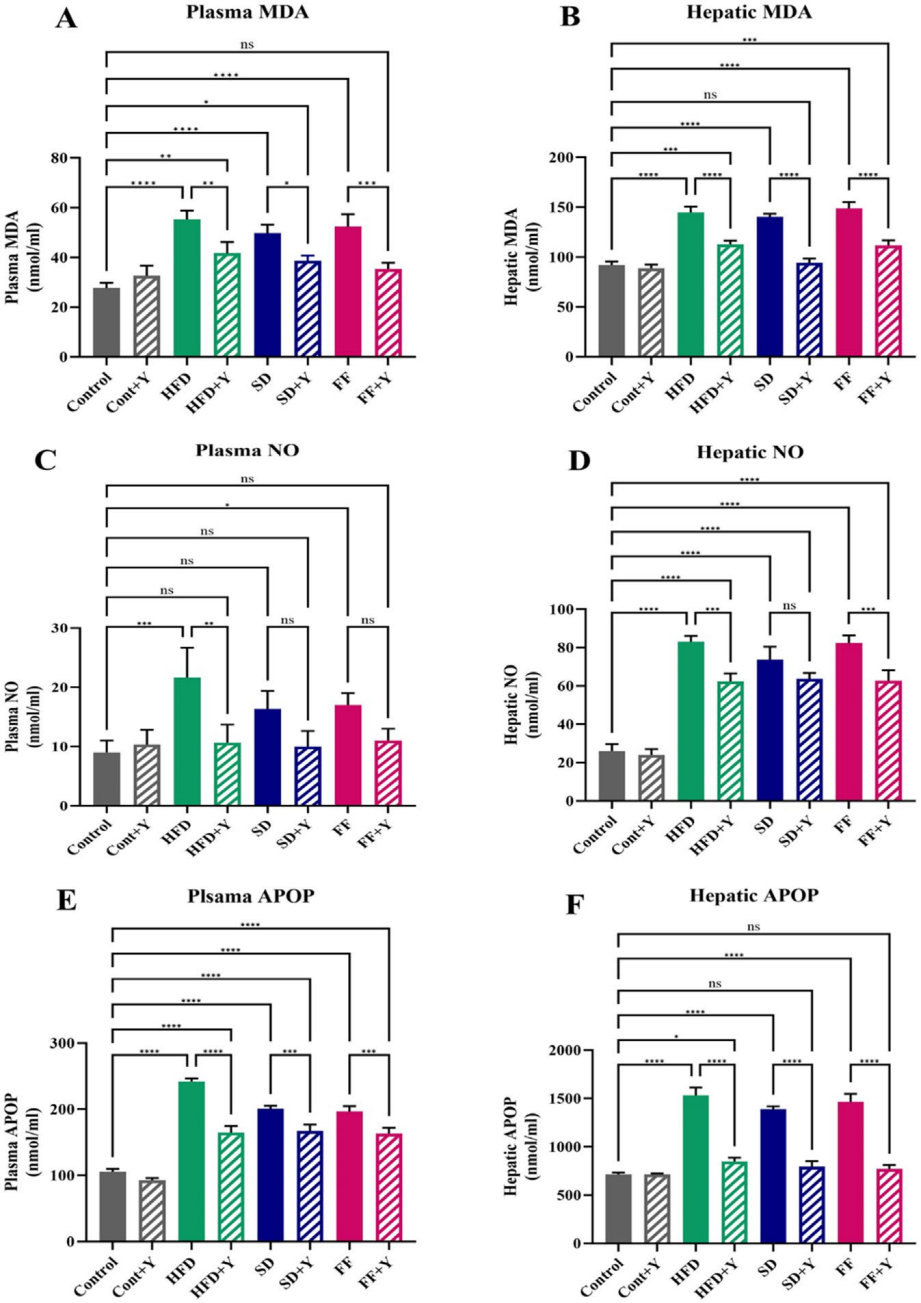
Effect of yogurt supplementation on liver antioxidant capacity and lipid peroxidation markers; (A) plasma MDA, (B) hepatic MDA; (C) plasma NO, (D) hepatic NO; (E) plasma APOP, and (F) hepatic APOP of HFD, SD, and FF‐treated mice. Control mice were provided with control diet and HFD, FF, and SD were provided with HF diet, FF diet, and Coca‐Cola. Yogurt was also provided to control + yogurt and HFD + yogurt, FF + yogurt and SD + yogurt groups. Data are presented as mean ± SD, *n* = 6. Statistical analysis was performed by One Way ANOVA with Šídák post hoc test using GraphPad Prism (10.2.3(403)). Statistical significance is considered as *p* < 0.05. Asterisk (****) marked data are significantly different at *p* < 0.0001 and (ns) denotes *p* > 0.05.

Table 4 depicts the impact of yogurt supplementation on hepatic lipid peroxidation and plasma levels in mice treated with control (chow diet), HFD, FF, and SD diets, respectively. Malondialdehyde (MDA) formation was used to measure lipid peroxidation that is caused by the lipid peroxidation of polyunsaturated FAs and is a biomarker for oxidative stress. In this experiment, MDA values in the liver and plasma were significantly (*p* < 0.05) elevated in HFD, FF, and SD‐treated mice as compared to control mice. However, HFD + yogurt, FF + yogurt, and SD + yogurt treated mice demonstrated reduced plasma MDA concentration compared to HFD, FF, and SD diet‐treated mice (Table 4) (Figure 4). On the other hand, APOP (advanced protein oxidation product) is released in oxidative stress condition from liver which is recognizes as the marker of oxidative damage to proteins. In this experiments, HFD, FF, and SD diet‐treated mice revealed elevated APOP level in plasma and liver tissues (Table 4) (Figure 4). Significantly, yogurt supplementation further reduced the rise of APOP level in HFD + yogurt, FF + yogurt, and SD + yogurt administered mice (Table 4) (Figure 4). In comparison to control mice, the plasma and liver homogenates of HFD, FF, and SD diet‐treated mice exhibited an increase in nitrate, a measure of nitric oxide. Supplementing with yogurt significantly restored the normal level of nitric oxide in both plasma and liver of HFD, FF, and SD diet‐administered mice (Figure 4). Hence, yogurt supplementation has a regulatory role on increase in antioxidant capacity and decreasing lipid peroxidation which helps to recover the damage or dysfunction of liver in hepatic steatosis condition.

**TABLE 4:** Effects of yogurt supplementation on various biochemical and oxidative stress markers in plasma and liver tissue of diet induced obese mice.

	Control	Ctrl + Y	HFD	HFD + Y	FF	FF + Y	SD	SD + Y
Plasma								
MDA (nmol/mL)	30.35	34.74	52.38	39.56	49.78	40.21	50.67	42.10
NO (nmol/mL)	8.76	9.57	17.89	10.27	15.98	12.10	14.67	9.32
APOP (nmol/mL)	105.79	85.34	211.57	176.19	197.33	179.56	180.45	162.45
Catalase (U/min)	16.33	18.67	5.21	10.21	7.40	13.11	8.23	11.67
GSH (μg/mg protein)	25.12	26.40	15.05	18.67	17.03	19.72	18.39	21.22
SOD (U/min)	326.67	315.56	162.98	287.64	174.04	249.34	180.45	230.33
Liver tissue								
MDA (nmol/mL)	91.51	92.11	139.92	104.45	141.90	110.67	133.71	105.02
NO (nmol/mL)	27.43	24.03	80.48	67.62	86.21	59.68	76.22	61.82
APOP (nmol/mL)	726.73	701.77	1445.37	806.33	1380.86	894.63	1347.33	807.34
Catalase (U/min/g)	16.28	14.88	7.56	13.22	9.50	14.87	10.03	15.93
GSH (μg/mg protein)	22.26	19.85	11.20	18.50	13.05	20.47	12.93	19.49
SOD (U/g tissue)	540.56	480.98	195.36	467.72	201.68	478.35	210.53	482.47
MPO (U/mg tissue)	2.14	2.78	8.20	5.83	7.20	5.52	7.88	5.56

*Note:* Control mice were provided with chow diet and HFD, FF, and SD group were provided with HF diet, fast‐food diet, and Coca‐Cola, respectively. Yogurt was also provided to control and other experimental groups. Data are presented as mean ± SD, *n* = 6. Statistical analysis was performed by One Way ANOVA with Šídák post hoc test using GraphPad Prism (10.2.3(403)). Statistical significance is considered as *p* < 0.05. Asterisk (****) marked data are significantly different at *p* < 0.0001 and (ns) denotes *p* > 0.05.

### 3.6 Effects of Yogurt Supplementation on Antioxidant Enzymes and GSH Status

**FIGURE 5:**
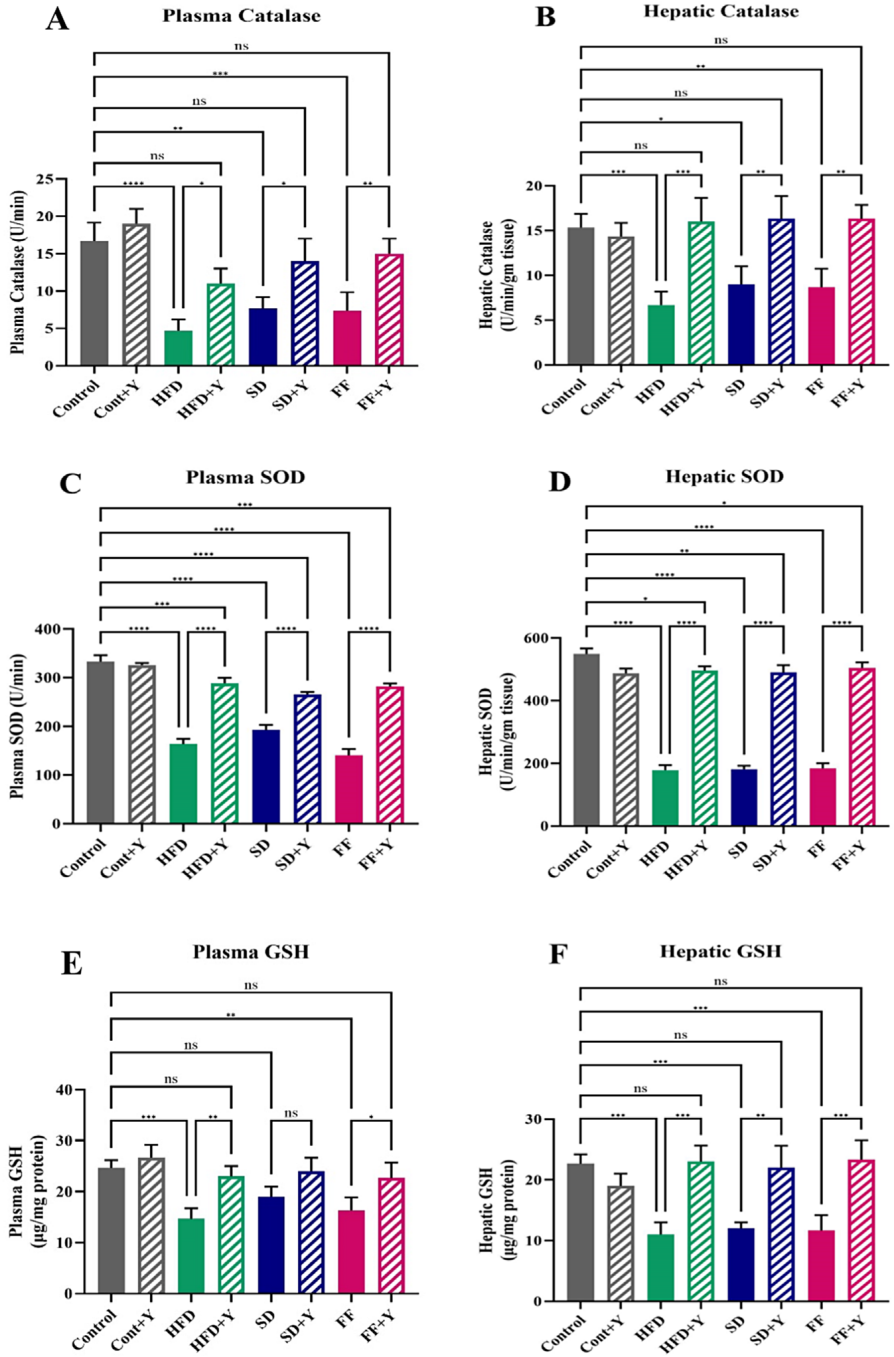
Effect of yogurt supplementation on Antioxidant enzymes and glutathione status; (A) plasma catalase, (B) hepatic catalase; (C) plasma SOD, (D) hepatic SOD; (E) plasma GSH, and (F) hepatic GSH of HFD, SD, and FF‐treated mice. Control mice were provided with control diet and HFD, FF, and SD were provided with HF diet, FF diet, and Coca‐Cola. Yogurt was also provided to control + yogurt and HFD + yogurt, FF + yogurt, and SD + yogurt groups. Data are presented as mean ± SD, *n* = 6. Statistical analysis was performed by One Way ANOVA with Šídák post hoc test using GraphPad Prism (10.2.3(403)). Statistical significance is considered as *p* < 0.05. Asterisk (****) marked data are significantly different at *p* < 0.0001 and (ns) denotes *p* > 0.05.

The impact of yogurt supplementation on GSH redox levels and antioxidant enzyme activities in the plasma and liver tissues of mice treated with HFD, FF, and SD diet, as well as control mice is demonstrated in Table 4. Superoxide dismutase (SOD) and catalase (CAT) are enzymes that helps to breakdown potentially harmful oxygen molecules in cells/tissues and thereby prevent damage to liver tissues. In this experiment, the HFD, FF, and SD diet treatment had a significant impact on the SOD and CAT activities in both the plasma and hepatic level where the activity is drastically downregulated in hepatic steatosis conditions. Interestingly, supplementing with yogurt with the experimental groups improved the changes in hepatic SOD and CAT activity significantly (*p* < 0.05) (Figure 5). Besides, GSH is the most important thiol reducing agents in liver involving in modulation in redox process. In high‐fat‐induced conditions like HFD, SD, and FF diet‐treated mice, the GSH level was significantly (*p* < 0.05) depleted in the both plasma and hepatic level as compared to the control mice. But, HFD + yogurt, FF + yogurt, and SD + yogurt treatment restored the enzymatic activity normal. In comparison to control mice, GSH levels in plasma and hepatic levels were restored by yogurt supplementation in HFD, FF, and SD diet‐treated mice which is very significant. So, comparing the HFD, FF, and SD diet challenge to the control mice also had an impact on the hepatic GSH levels (Figure 5). Hene, this result strongly evident that yogurt is an important factor to normalize the abnormal functions of different antioxidant enzymes of liver in hepatic steatosis condition.

### 3.7 Effect of Yogurt Supplementation on the Lipid Profiles of HFD, SD, and FF Diet‐Fed Mice

**FIGURE 6:**
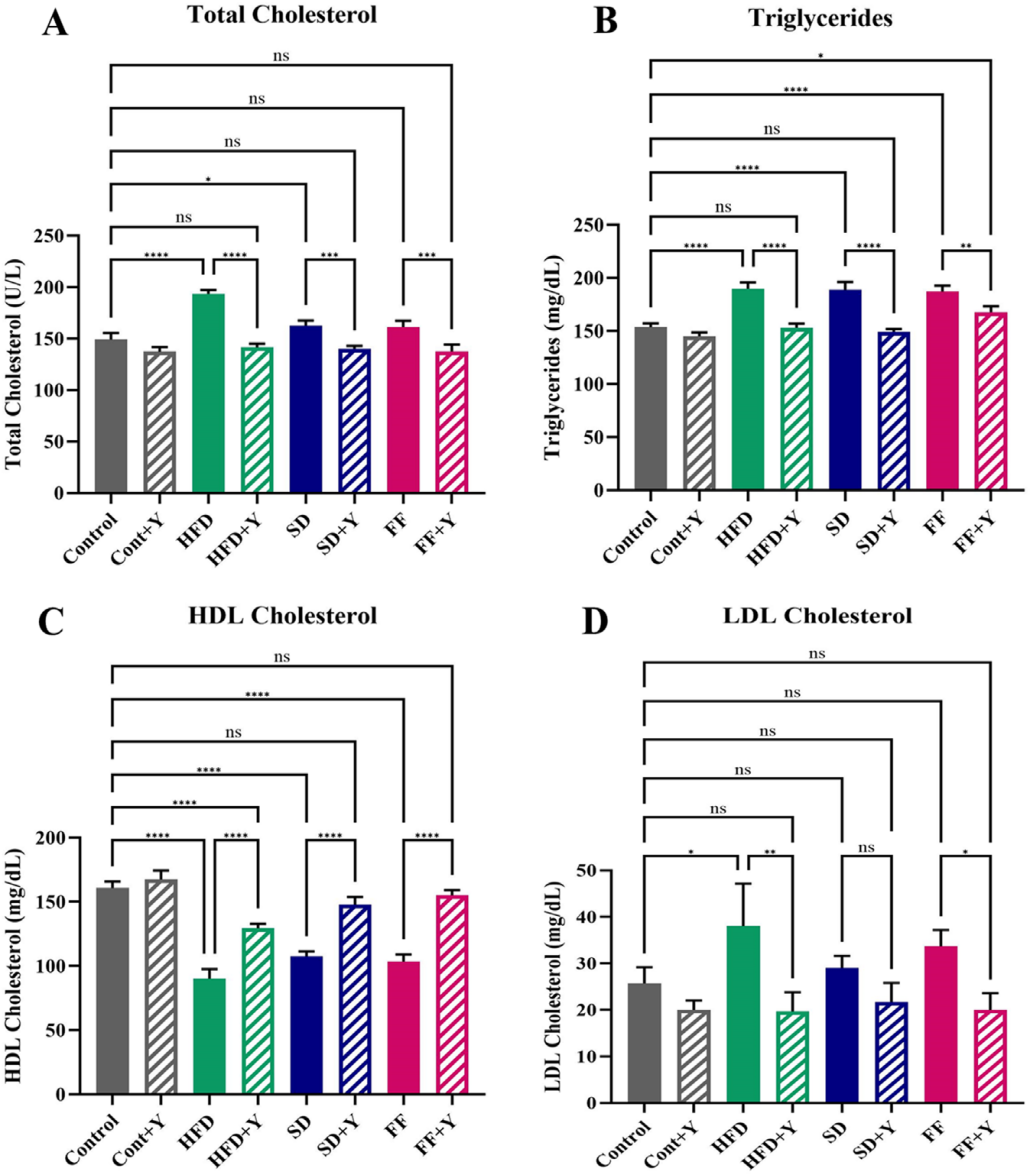
Impact of yogurt supplementation on the lipid profiles of HFD, FF, and SD diet‐fed mice; (A) total cholesterol, (B) triglycerides, (C) HDL, and (D) LDL of HFD, SD, and FF‐treated mice. Control mice were provided with control diet and HFD, FF, and SD were provided with HF diet, FF diet, and Coca‐Cola. Yogurt was also provided to control + yogurt, HFD + yogurt, FF + yogurt, and SD + yogurt groups. Data are presented as mean ± SD, *n* = 6. Statistical analysis was performed by One Way ANOVA with Šídák post hoc test using GraphPad Prism (10.2.3(403)). Statistical significance is considered as *p* < 0.05. Asterisk (****) marked data are significantly different at *p* < 0.0001 and (ns) denotes *p* > 0.05.

To assess the lipid‐lowering impact of yogurt supplementation on HFD, FF, and SD diet‐fed mice, we evaluated the levels of total cholesterol and triglycerides in their plasma. In Figure 6, the lipid profile of HFD, FF, and SD diet‐treated mice along with the yogurt supplement is shown. In our present investigation, the mice fed with the HFD, FF, and SD diets had significantly higher plasma triglyceride and total cholesterol levels (*p* < 0.05) than the control group which strongly indicates the liver turning into fatty liver condition. This effect is might be due to the metabolic dysfunction of lipids in hepatocytes as well as increased lipid droplet biogenesis in hepatocytes and other hepatic cells. Here, the genes responsible for triacylglycerols (TAGs) and cholesterols metabolism might be dysregulated that results in hepatic steatosis. Supplementing with yogurt led to a significant decrease in plasma triglyceride and total cholesterol levels in HFD, FF, and SD diet‐fed mice (Figure 6). Furthermore, when comparing the HFD, FF, and SD diet‐fed mice to the control mice, the low‐density lipoprotein (LDL) cholesterol level was also considerably higher (*p* < 0.05) (Figure 6). But, when yogurt was added to HFD, FF, and SD diets, the amount of plasma LDL cholesterol level dropped dramatically (*p* < 0.05). Additionally, compared to control mice, the high‐density lipoprotein (HDL) cholesterol level was significantly reduced in the HFD, FF, and SD diet‐fed mice (*p* < 0.05). Nevertheless, yogurt was identified as a novel therapeutic agent that play an important role for up regulation of lipid metabolism and decreased the lipid droplet formation rate. However, the lipid profiles of the yogurt supplemented control mice did not display any significant change compared to wild‐type, rather showed similar pattern (Figure 6). This clearly indicate that high fat diet had a predominant role in producing fat molecules with excessive accumulation of lipids in hepatocytes and dysregulation of its metabolism. Hence, yogurt might be used as targeted agents for the treatment of fatty liver disease as it decreases the magnitude of this disease.

### 3.8 Histological Analysis of Liver Tissues

**FIGURE 7:**
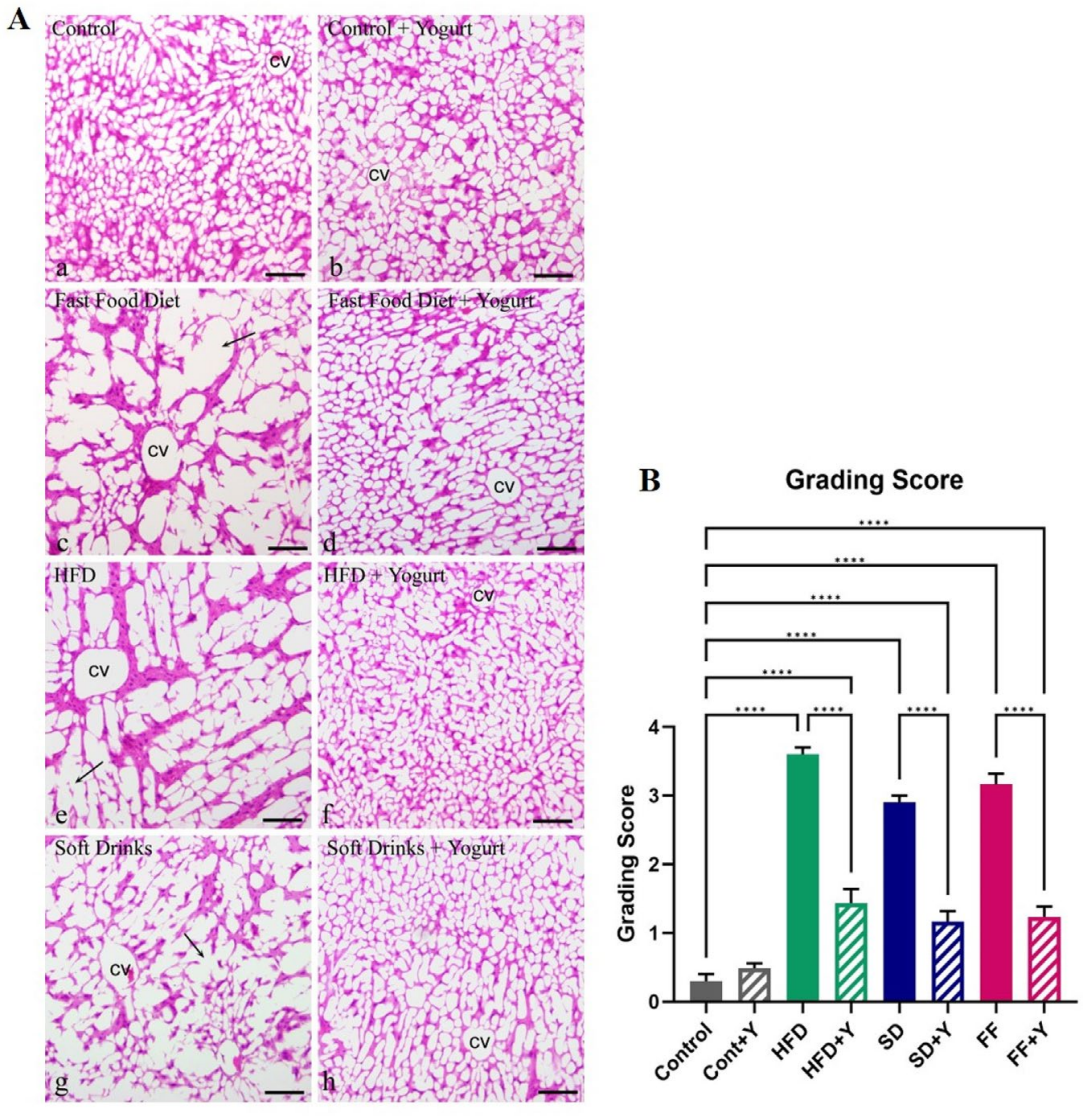
Impact of yogurt supplementation on hepatic architecture in HFD, FF, and SD diet‐fed obese mice: (A) Histomicrographs showing the changes in liver texture in HFD, FF, and SD diet, and their recovery in yogurt (y) supplementation in mice. Sections were stained with hematoxylin and eosin stains. Control mice were provided with chow diet, and HFD, SD, and FF groups were provided with 60% high fat diet, Coca‐Cola, and fast‐food diet, respectively. Yogurt was supplied to control and other experimental groups as therapeutic agents. Control (a) and control + yogurt (b) did not display any lesions and necrosis in liver tissues and showed normal histological architecture. FF (c); HFD (e); and SD (g) treated groups showed aberrated liver texture with high accumulation of lipid droplets in liver tissues and inflammation. But, HFD + Y, SD + Y, and FF + Y‐treated groups displayed improvement in reducing fat accumulation and inflammation of hepatic tissues. So, yogurt has a significant role on lowering lipid accumulation as well as lipid droplet biogenesis down regulation. (B) Grading Score. Data are presented as mean ± SD, *n* = 6. Statistical analysis was performed by One Way ANOVA with Šídák post hoc test using Graphpad Prism (10.2.3(403)). Statistical significance was considered as *p* < 0.05. Asterisk (****) marked data are significantly different at *p* < 0.0001, and NS denotes *p* > 0.05. Abbreviations: HFD, high‐fat diet; cv, central vein; arrows indicate aberrated liver texture with high accumulation of lipid droplets in hepatocytes; scale bar 100 μm.

High fat diets had a predominant role in generating higher levels of inflammatory cell infiltration and hepatic lipid accumulation. Histomicrographs showed the changes in liver texture in HFD, FF and SD diet, and their recovery in yogurt (y) supplementation in mice (Figure 7). Control mice were provided with normal chow diet, and HFD, SD, and FF groups were provided with 60% high fat diet, Coca‐Cola, and fast‐food diet, respectively. Yogurt was supplied to control and other experimental groups as therapeutic agents. Control and control+ yogurt supplemented group did not display any lesions and necrosis in liver tissues and showed normal histological architecture. Compared to control mice, control+ yogurt mice showed no alterations in tissue morphology, inflammatory cell infiltration, or macro‐vesicular steatosis. On the other hand, FF; HFD and SD treated groups showed aberrated liver texture with high accumulation of lipid droplets in liver tissues and inflammation (Figure 7). But, HFD + Y, SD + Y, and FF + Y‐treated groups displayed improvement in reducing fat accumulation and inflammation of hepatic tissues. In fact, yogurt supplementation decreased macro‐vesicular steatosis and portal inflammation in HFD, SD, and FF diet‐fed mice So, yogurt has a significant role on lowering lipid accumulation as well as lipid droplet biogenesis down regulation. Moreover, grading score was analyzed by One Way ANOVA with Šídák post hoc test using GraphPad Prism (10.2.3(403)), which showed a significant difference between control and the experimental groups. However, excess lipid was deposited in different tissues of liver fed with HFD, FF, and SD diet, which results in fatty liver disease. So, yogurt might be used as a therapeutic agent for the treatment of NAFLD.

These findings were also supported by lipid droplets morphology analysis by H and E staining with Oil red O. Fast‐food, HFD, and sSD‐treated mice displayed fatty liver phenotype and increases LD biogenesis/accumulation (Figure S1). Normal mice liver contained small size lipid droplets molecules in hepatocytes all over the liver. While, HFD, FF and SD‐treated mice liver showed large amount of lipids molecules accumulation with big size of lipid droplets in hepatocytes. But yogurt treatment downregulates the formation of lipid molecules and accumulation of lipid droplets in hepatocytes and give rise to a normal phenotype of liver. Hence, yogurt plays a significant role in downregulating the formation of lipid droplet and thereby act as a therapeutic agent for fatty liver disease.

### 3.9 Analysis of Lipid Molecules Purified From Lipid Droplets of Liver Tissues and Its Densitometry Analysis by Image J

**FIGURE 8:**
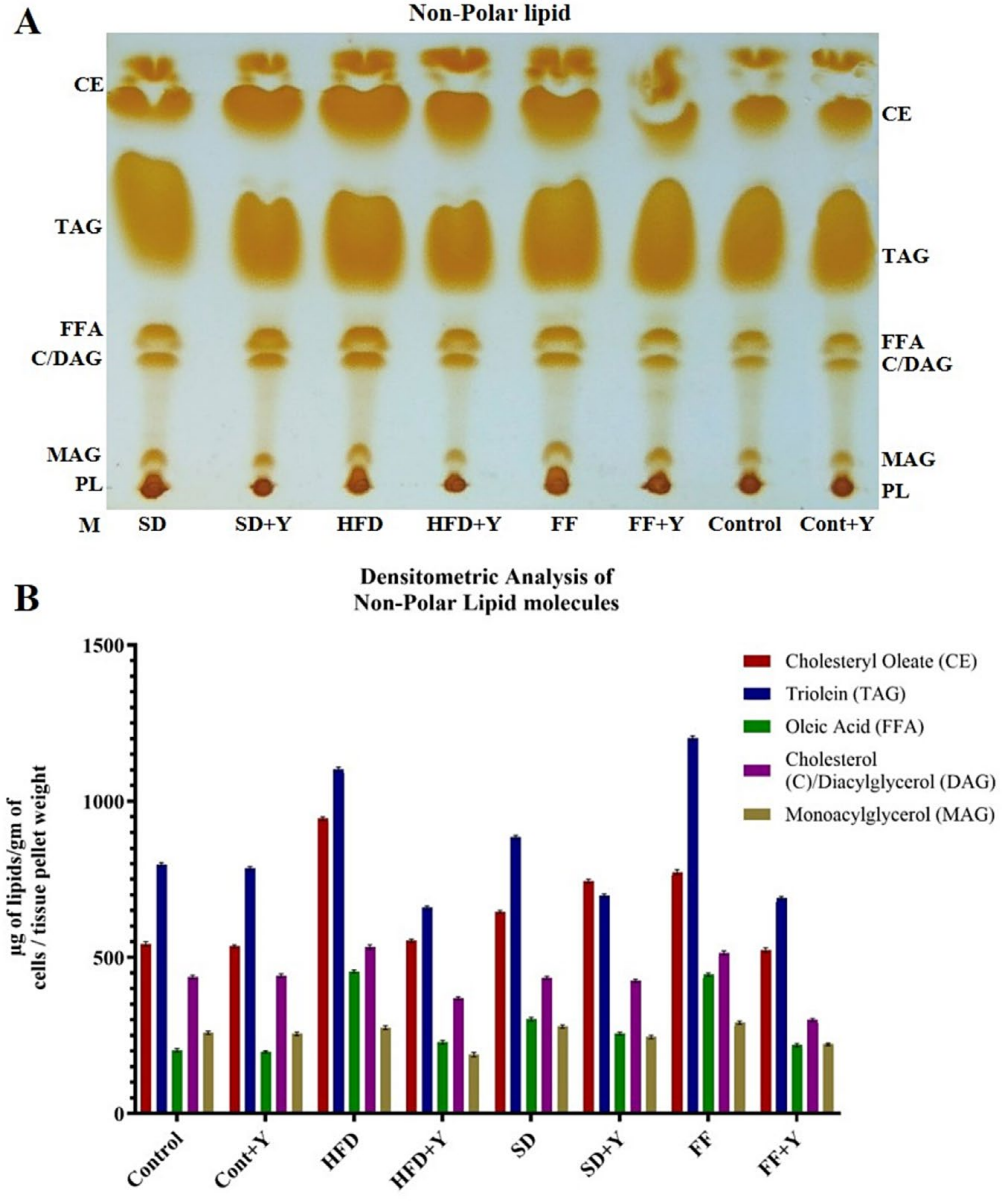
Increased amount of lipids accumulation in HFD, SD and FF treated group, while yogurt supplementation decrease drastically. Panel‐A: TLC analysis of non‐polar lipid. Lipids were extracted from the LDs of different groups of liver tissues with same wet weight of cells. For non‐polar lipids, *n*‐hexane/diethyl ether/acetic acid (80:20:1) was the developing solvent. CE, cholesterol ester; TAG, triacylglycerol; FFA, free fatty acids; C, cholesterol; and PL, polar lipid; HFD, FF, and SD‐treated groups showed a higher amount of CE, TAG, FFA, and cholesterol than the control and yogurt applied group. *R*_*f*_ values of 0.069, 0.280, 0.340, 0.615, and 0.858 indicating polar lipid, cholesterol, free fatty acid, triacylglycerol, cholesterol ester, respectively. Panel‐B: Densitometric analysis of non‐polar lipid molecules.

**FIGURE 9:**
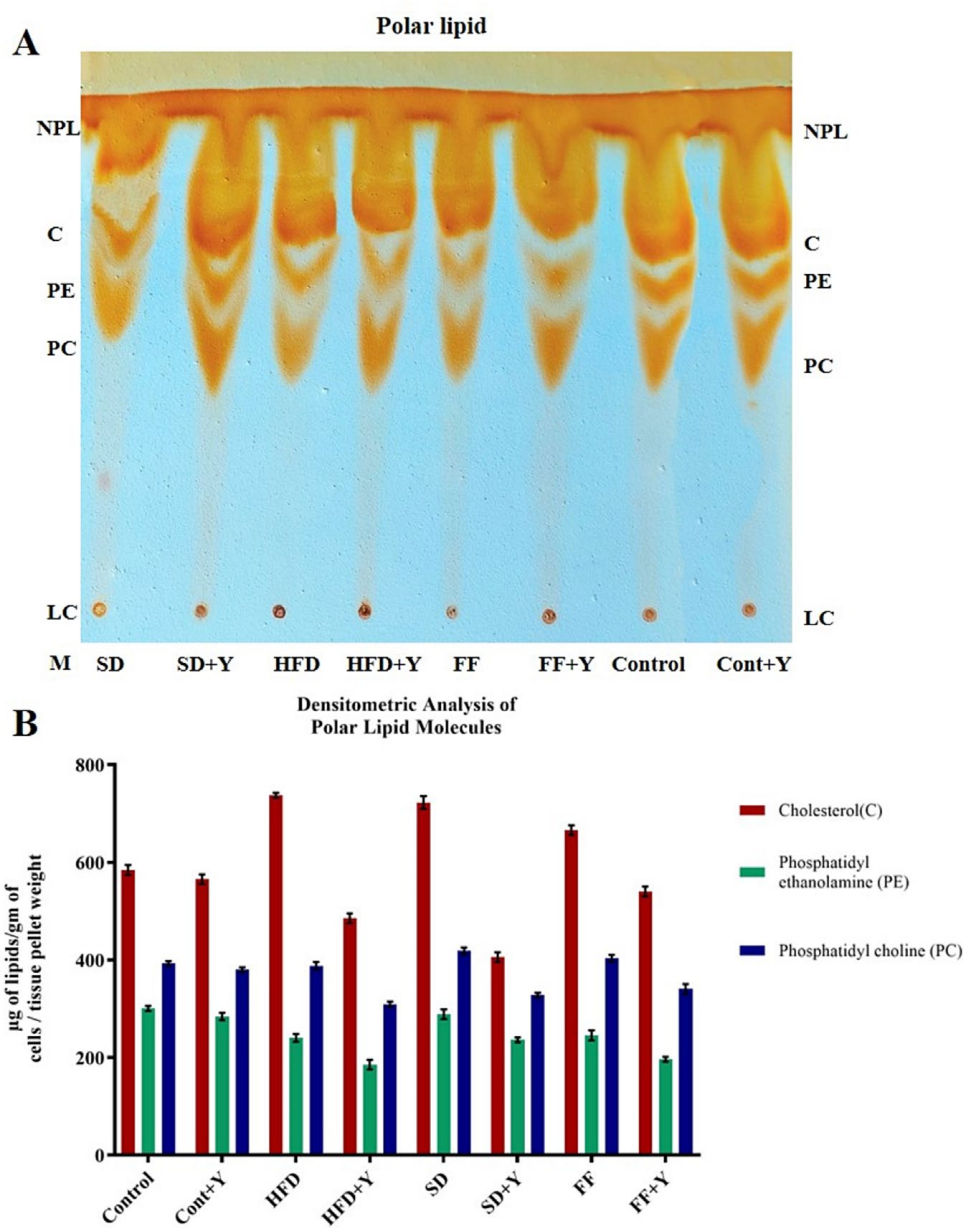
Decreased rate of polar lipid accumulation in high‐fat‐induced conditions. Panel‐A: TLC analysis of polar lipid: The solvent system was chloroform/methanol/acetone/acetic acid/water (65:35:10:5:4) as developing solvent. NPL, non‐polar lipid; C, cholesterol; PE, phosphatidyl ethanolamine; PC, phosphatidyl choline; and LC, lyso‐lecithin. Panel‐B: Densitometric analysis of polar lipid molecules were analyzed by Image J. software.

Any defects in normal homeostasis process of lipid metabolism in liver causes non‐physiological deposition of TAGs in liver cells/tissues. Excessive accumulation of lipids in lipid droplet is the central to the pathogenesis metabolic diseases like fatty liver disease. To investigate this issue, lipids were extracted and purified and characterized by different biochemical and chromatographic techniques. Composition of different lipid molecules was separated and analyzed by TLC and subsequently amount was measured by using Image J software. TLC analysis of non‐polar lipid: Lipids were extracted from the LDs of different group of mice liver with the same wet weight of cells. *n*‐hexane: diethyl ether: acetic acid (80:20:1) was the developing solvent. Significant amount of increased lipids was found in the experimental group expressing fatty liver disease (NAFLD). HFD, FF, and SD diet‐treated groups showed a higher amount of CE, TAG, FFA, and cholesterol than the control group (Figure 8). But, after yogurt supplementation with vitamin B complex, these lipid parameters were downgraded as like as control group which was very significant. This might be due to the up regulation of lipid droplet biogenesis in the high‐fat‐induced condition in the experimental group or due to the dysregulation of lipid metabolism or dysfunction of metabolic enzymes. Nevertheless, yogurt supplementation to the experimental group decreases the lipid droplet biogenesis and increase the metabolic rate of the enzymes and thereby decrees the fat accumulation in the liver tissues and result in the protective role of formation of fatty liver diseases. Hence, yogurt supplementation plays an important role for decreasing the magnitude of the fatty liver disease and might be used as important therapeutic agents for this disease. *R*_*f*_ values of 0.069, 0.280, 0.340, 0.615, and 0.858 indicating polar lipid, cholesterol, free fatty acid, TAGs, cCE, respectively. Densitometric analysis of non‐polar lipid molecules were measured and analyzed by Image J method and the results support the above findings which is very supportive. Moreover, TLC analysis of polar lipid from the liver tissues was performed and displayed that yogurt with vitamin B complex treated as therapeutics contained higher amounts of polar lipids that is phosphatidyl choline (PC), cholesterol (C), and phosphatidyl ethanolamine (PE) as compared to control as well as HFD, FF, and SD diet‐treated groups (Figure 9). Chloroform: methanol: acetone: acetic acid: water (65:35:10:5:4) was used as developing solvent system. Densitometric analysis of polar lipid molecules were also measured and analyzed by Image J method with the same line of findings. So, the effects of yogurt as therapeutics agent had an effective protective role against fatty liver disease which magnifies its important function in preventing fatty liver disease.

## 4 Discussion

Globally, there is an increase in the health complications associated with obesity and NAFLD in many societies. Eating a diet heavy in fat and energy is thought to be a major causative factor to the development of these problems. Liver plays central role in lipid metabolism in hepatocytes; like, lipid uptake, oxidation, esterification and fatty acid secretion. In liver, 15%–30% of FAs are originated from diet, up to 30% are by *de‐novo* lipid biogenesis and release of FAs and other lipid molecules from adipose tissues during starvation (Mashek 2013). Moreover, in intestinal lumen, enterocytes take up the FAs which are broken down from TAG and temporarily stored in lipid droplets or a complex of TAG and CEs in chylomicron for secretion (Hussain 2014). Subsequently, TAG is then transported to peripheral tissues from chylomicron and other remaining lipid molecules are delivered to liver (Nestel, Havel, and Bezman 1962). According to the present study, mice fed a HFD developed oxidative stress in the liver, dyslipidemia, and glucose intolerance. Furthermore, in high‐fat‐induced conditions, mice fed a HFD, treatment with yogurt supplementation reduced plasma lipid levels and prevented glucose intolerance. Supplementing with yogurt also restored the cellular antioxidants, preventing oxidative stress and reducing lipid peroxidation in the hepatocytes and plasma.

Inequities of the normal homeostasis process of lipid metabolism in liver causes non‐physiological deposition of TAGs in hepatocytes (Kawano and Cohen 2013). In liver, FAs can be converted to TAG and CE to be secreted as very low‐density lipoprotein (VLDL) units (Tiwari and Siddiqi 2012). Although, the composition and mechanism of formation of LDs and VLDL is not similar. The majority of clinical patients and experimental models exhibit oxidative stress is due to eating a HFD (Mohammadi et al. 2015; Yadav, Jain, and Sinha 2008). The current study demonstrates that mice fed a HFD, FF, and SD have increased lipid peroxidation levels and its derivatives malondialdehyde, nitric oxide, and advanced protein oxidation products (APOP). The augmented indices of oxidative stress may be linked to the observed reduction in SOD and catalase activity. These findings are in line with other research that suggested animals on a HFD may have impaired tissue antioxidant defenses (Ulla et al. 2017; Lee et al. 2009). In our study, yogurt supplementation reduced oxidative stress as well as restored the tissue antioxidants in mice fed a HFD, FF, and SD. Supplementing with probiotics may strengthen the antioxidant defenses in oxidative stressed tissues. However, LAB‐rich pickled Chinese cabbage supplemented with a HFD rises SOD and GSH‐Px activity in ICR mice (Gao et al. 2011). Additionally, probiotics have a favorable modulatory effect on free radical metabolism by upregulating antioxidant enzyme activity and lowering nitric oxide and malondialdehyde (MDA) concentrations (Chen, Gong, and Xu 2013; Uskova and Kravchenko 2009).

Excessive TGs accumulation in LDs results from the increased biogenesis of TAG combined with LD synthesis and growth as well as downregulation of lipid catabolic pathways such as fatty acid oxidation and impaired VLDL/TAG secretion (Kawano and Cohen 2013). Mice that were fed a diet high in fat, FF, and SD showed elevated levels of both plasma glucose and plasma lipids, such as triglycerides and cholesterol. The rise in lipid profiles and glucose levels is caused by the presence of saturated fat molecules in the HFD (Timmers et al. 2011). Previous research has shown that rats fed in high‐fat‐induced conditions have higher glucose levels in blood (Akiyama et al. 1996). Additionally, to developing glucose intolerance, rats given a HFD were also unable to use glucose to restore homeostasis following a glucose challenge and metabolic imbalance. Indeed, lipopolysaccharide (LPS) found in gram‐negative bacteria cell wall has the ability to enter the gut because a high fat diet can cause the intestinal mucosa to leak (Cani et al. 2008). It has been suggested by researchers that inflammation and endotoxemia may be caused by bacterial LPS (Naito et al. 2011). Additionally, intestinal permeability may make it easier for bacterial fragments to enter the body, engaged with the Toll‐like receptor to activate immunity, and result in resistance of insulin activity and hyperglycemia (Amar et al. 2011). Nevertheless, some species of Lactobacilli have the ability to strengthen the epithelial barrier, which can assist with preventing inflammation and hyperglycemia caused by LPS (Hummel et al. 2012). In this experiment, the glucose consumption was enhanced by the addition of yogurt, as demonstrated by the OGTT results, and a prior experiment supports these findings (Yeon et al. 2015). In this research, yogurt supplementation also inhibited the accumulation of epididymal adipose tissue in mice fed with FF, HFDs, and SD as diet. Previous research has shown that yogurt dramatically reduces the risk of metabolic syndrome, which includes obesity (Spence, Cifelli, and Miller 2011; Marette and Picard‐Deland 2014).

On the other hand, the elevated plasma activity of the enzymes AST, ALT, and ALP shows that the oxidative stress in tissues also causes hepatocyte injury in mice fed HFD, FF, and SD. These enzymes are thought to be indicators of hepatic dysfunction. These enzymes are typically transferred to the plasma as a result of hepatocyte injury. In hepatic steatosis conditions, ALT, AST, and ALP levels become high due to abnormal function of the liver due to inflammation or hepatic injury. ALT is the most widely used single liver chemistry employed in the diagnosis and the most important tools commonly used for detecting NAFLD (Draijer et al. 2019), which was decreased in the case of yogurt supplementation. This might be due to the metabolic activity of probiotic effects of yogurt that normalize the ALT activity, which is important for normal function of liver. Yogurt is helpful in reducing intestinal and liver inflammation and function in decreasing hepatic injury to maintain normal liver condition. This helpful activity of yogurt to these enzymes thereby reduces the magnitude of fatty liver disease NAFLD. This is a very significant finding of the identifications of factors causing the liver disease as well as its therapeutic agents. In high‐fat‐induced conditions, it exhibited higher levels of cholesterol and plasma triglycerides, which eventually led to the progress of lipotoxicity and buildup of lipids in the liver tissues and cells (Ulla et al. 2017). NAFLD is the term for deposition of fat molecules in the hepatocytes that eventually leads to steatosis. A “two hit” idea has been put up to elucidated how the progression of NAFLD is brought on by HFD induction (Haga et al. 2015). According to Esposito (Esposito et al. 2009), the key elements of this “two hit” approach are storage of fat molecules, resistance of insulin, oxidative stress, and inflammation of histological texture of hepatocytes as the root causes of disease. In this study, mice given a diet high in fat (60%), FF, or SD displayed accumulation of lipids and an increase in inflammatory cells infiltration into the liver. Moreover, in high‐fat‐induced conditions, the mice livers also showed an increase in myeloperoxidase activity, a marker of invading cells, primarily neutrophils. Probiotics have been shown to be helpful in reducing intestinal and liver inflammation (Mach 2006; Loguercio et al. 2002). Moreover, it was reported that 4 weeks of yogurt supplementation reduces the lipid profiles in human subjects (Xiao et al. 2003). Additionally, a probiotic mixture reduces inflammatory indicators and improves increased lipid profiles in NAFLD patients (Muzafar and Amin 2017). It was clearly evident through histopathological observations that excessive deposition of fat molecules can create lesions and necrosis in liver tissues and destroy the normal texture of hepatocytes. In high‐fat‐induced conditions, liver cells become aberrated along with alterations in tissue morphology, inflammatory cell infiltration, or macrovesicular steatosis. Liver histology in this current investigation suggested that yogurt supplementation might have an impact on reducing inflammation and hepatic steatosis in mice fed a HFD, FF, and SD and restore normal histological architecture.

In line with the formation of NAFLD, the mucosal barrier of the intestinal epithelium controls inflammation caused by bacteria or other allergens in the intestine. The GIT is home to certain LAB. To some extent, these bacteria seem to have positive impacts on modulating the immune response, cytokine production, intestinal permeability, and inhibiting the proliferation of harmful bacteria (Yu et al. 2012). In rats and humans fed a HFD, supplementation of yogurt as probiotics decreases cholesterol and triglyceride levels in plasma (Kumar et al. 2012). Nevertheless, our findings are also consistent with the previous results and demonstrated a drop in triglyceride and cholesterol levels in plasma. Probiotic‐induced bile acid deconjugation and binding of cholesterol have also been suggested as potential pathways for lowering the action of cholesterol by the probiotic supplementation (Ooi and Liong 2010). The bile acid conjugates can be hydrolyzed by Lactobacillus species, rendering it insoluble in water and ultimately excreted in the stools (Begley, Hill, and Gahan 2006). Absorption of FAs is declined by this action from the gut by preventing the saponification of FAs and boosts the production of bile acid from cholesterol over homeostasis (Ooi and Liong 2010). In line with this, in NAFLD, sarcopenia is associated significantly with liver fibrosis in high‐fat‐induced obese conditions. Sarcopenia,a severe condition common to various chronic liver diseases caused by gut microbiota, is a major health problem (Zhou et al. 2023). Our study of fatty liver disease was mainly conducted using mice as a model and needs to be a human trial for use as a therapeutic agent. Another study reported that the animal model of NAFLD does not completely mirror the human disease when translating their results (Zheng et al. 2023).

In fine, this research showed that yogurt supplementation is an alternative strategy for treating complications related to fatty liver disease as well as obesity. In high‐fat‐induced conditions, lipid molecules are accumulated in LDs that enhance its biogenesis process and growth. Simultaneously, dysregulation of lipid metabolism causes deposition of TAG and CEs in hepatocytes that results hepatic steatosis. Furthermore, consuming FF, SDs, and a HFD may change the typical environment in the gut, which can be altered by yogurt supplementation as probiotic‐rich factors. Thus, yogurt might be used as a significant therapeutic tool for combating against fatty liver disease. This finding definitely will help to manage and treat fatty liver disease and serve mankind.

## Author Contributions

S.H., M.A.I.A., and M.M. managed the animal handling and feeding. M.M. and S.K. performed the OGTT and collected data. S.H., S.K., M.M., M.E.H., M.A.I.A., and Y.A. carried out the animal sacrifices and collected samples. S.H., M.A.I.A., M.E.H., M.M., and S.K. analyzed the data. R.G. carried out histopathology of liver tissue. M.A.I.A. conducted all the data analysis. S.H. and M.A.I.A. wrote the manuscript. S.H. designed the study, and trained the team for research. M.B., K.S.A. writing, reviewing, and eddying. All authors reviewed and approved the final version of the manuscript.

## Ethics Statement

Ethical approval was obtained from the Ethics Committee of Institute of Biological Sciences, University of Rajshahi, Bangladesh (249(35)/320/IAMEBBC/I.B.Sc.).

## Conflicts of Interest

The authors declare no conflicts of interest.

## Supporting information


**FIGURE S1.** Fast‐food, HFD, and soft drink‐treated mice displayed fatty liver phenotype and increases LD biogenesis/accumulation.
**FIGURE S2.** Fast‐food, HFD, and soft drink‐treated mice showed much weight gain, while yogurt treatment reduce it significantly.

## Data Availability

Data are available to the corresponding author upon reasonable request.
