# Supplementary material for: Yogurt Supplementation Can Ameliorate Fatty Liver Diseases and Metabolic Syndrome in High Fat‐Induced Conditions in Mice
Source: Food Sci Nutr. 2024 Dec 5;13(1):e4650. doi: 10.1002/fsn3.4650 (PMC11716991; doi:10.1002/fsn3.4650)
Supplement: Supplementary file 1 — FIGURE S1. Fast‐food, HFD, and soft drink‐treated mice displayed fatty liver phenotype and increases LD biogenesis/accumulation. FIGURE S2. Fast‐food, HFD, and soft drink‐treated mice showed much weight gain, while yogurt treatment reduce it significantly. [file FSN3-13-e4650-s001.docx]

**FIGURE S1 (supplementary). Fast-food, HFD and Soft drinks treated mice displayed fatty liver phenotype and increases LDs biogenesis/accumulation.** Hematoxylin and eosin stains of the mice livers of, a) Control, b) Control + Yogurt; c) Fast-food treated, d) Fast-food+ Yogurt; e) HFD treated, f) HFD + Yogurt; g) Soft drinks treated, h) Soft drinks + Yogurt. Frozen sections of livers were stained with by Oil red O in order to analyze the morphology and accumulation of lipids/LDs. Magnification, ×400. Control mice liver contained small lipid droplets molecules in hepatocytes all over the liver. While, HFD, Fast-food and Soft drinks treated mice liver showed large amount of lipids molecules accumulation with big size of lipid droplets in hepatocytes. But yogurt treatment downregulates the formation of lipid molecules and accumulation of lipid droplets in hepatocytes and give rise to a normal phenotype of liver.

**FIGURE S2 (supplementary). Fast-food, HFD and Soft drinks treated mice showed much weight gain, while yogurt treatment reduce it significantly.** During the experiment feeding period (98 days), body weight, food consumption, and water intake were recorded daily. The typical chow diet for control mice consisted of wheat, rice polishing, wheat bran, and fish meal, with a caloric value of roughly 25% proteins, 60% carbohydrates, and 15% fat. For experimental mice, High Fat Diet (Custom diet for lab animal, 60% fat, SYNERGY BIO, China) was administered. High-Fat (HF) diet included normal foods such as sugar, condensed milk, and beef tallow, and its calorie composition was roughly 14% proteins, 37% carbs, and 60% fat. Fast Food contains normal food with 30% cheese, 20% mayonnaise and 10% tomato slouch. Soft drinks contain 2ml coca-cola daily intake by the gavage.

**
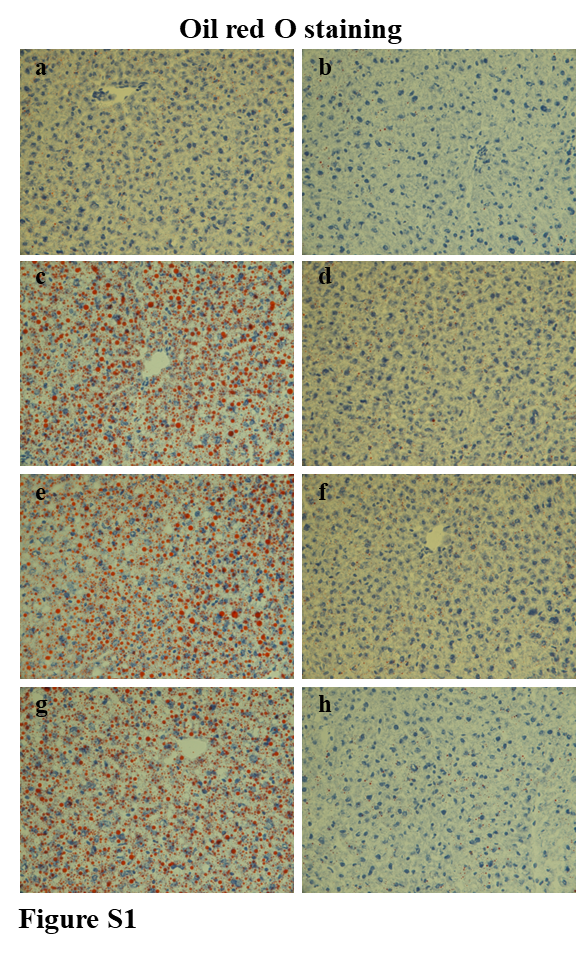
**

**
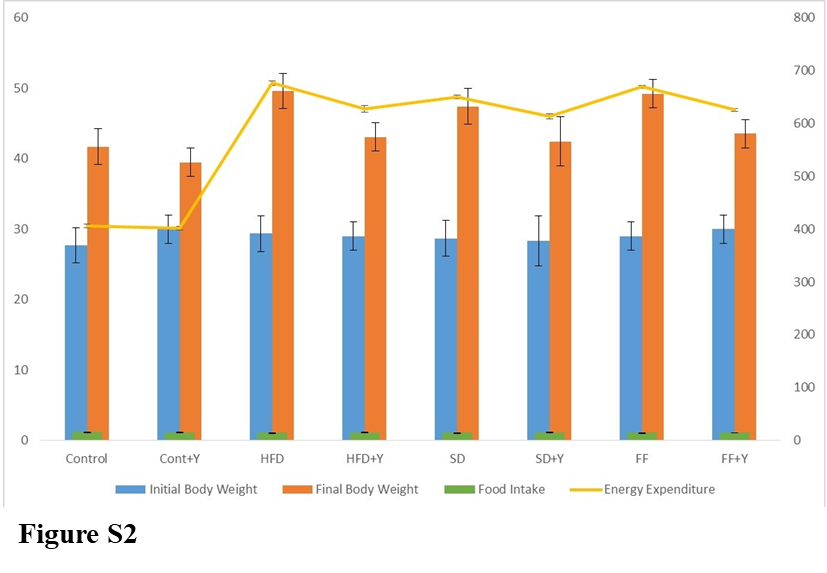
**
